# Global burden of stroke attributable to secondhand smoke in 204 countries and territories from 1990 to 2019: analysis of the global burden of disease study

**DOI:** 10.3389/fneur.2024.1320033

**Published:** 2024-01-26

**Authors:** Xinyue Yang, Jiayi Sun, Wenjuan Zhang

**Affiliations:** Department of Cardiovascular Medicine, Tianjin Medical University General Hospital, Tianjin, China

**Keywords:** stroke, secondhand smoke, global burden, sociodemographic index, health policy and planning

## Abstract

**Background:**

Secondhand smoke (SHS) continues a significant public health concern globally. This study aimed to assess the global burden of stroke attributable to SHS exposure during 1990–2019.

**Methods:**

This analysis utilized data on stroke morbidity and mortality from the Global Burden of Disease (GBD) 2019 study covering 204 countries and territories. We estimated stroke burden indicators attributable to SHS exposure, including age-standardized mortality rate (ASMR) and age-standardized disability-adjusted life-year rate (ASDR), stratified by age, sex, region, and stroke subtype.

**Results:**

In 2019, global SHS exposure accounted for 2.01 [95% uncertainty interval (UI): 1.49–2.58] million stroke mortality. The ASMR and ASDR were 2.5 (95% UI: 1.9–3.2) and 61.5 (95% UI: 46–78.8) per 100,000 population, respectively. The disease burden was higher among women than men and higher among the elderly than younger populations. Intracerebral hemorrhage and ischemic stroke had a more significant burden than subarachnoid hemorrhage. From 1990 to 2019, the ASMR and ASDR declined [estimated annual percentage change: −2.08 (95% CI: −2.21% to −1.95%) and −2.08% (95% CI: −2.19% to −1.97%) for each], but the absolute number of mortalities increased along with population growth. Substantial disparities existed across regions and sociodemographic groups.

**Conclusion:**

Despite declining ASMR and ASDR over time, the absolute number of stroke deaths attributable to SHS continued to rise globally, imposing a considerable stroke burden worldwide. These findings can inform targeted interventions and policies aimed at SHS control.

## Introduction

Secondhand smoke (SHS) represents a significant public health issue globally, contributing to over 800,000 premature deaths annually as part of the global tobacco epidemic (1). There is substantial evidence exists that SHS inhalation can increase the risk of major diseases, including cardiovascular disease, cancer, and respiratory illnesses (2–4). Notably, the health hazards from SHS exposure may be even more severe than from direct smoking, owing to the higher concentrations of toxic constituents in SHS (5). Although previous studies have validated the elevated stroke risk associated with SHS exposure, a comprehensive assessment quantifying the global disease burden of stroke specifically attributable to SHS has not yet been performed.

Stroke remains a leading cause of death and disability, with approximately 13 million cases occurring worldwide yearly (6). Exposure to SHS shows evidence potentially related to damage of endothelial cells and increased thrombosis, which may elevate the risk of stroke (7, 8). Controlling SHS exposure is expected to reduce the incidence of stroke, though data varies between countries and regions. Therefore, it is crucial to comprehensively assess the disease burden of stroke caused by SHS in various countries, which can support policies for SHS prevention and control.

Utilizing data from the Global Burden of Disease (GBD) study, we conducted the first comprehensive evaluation of the stroke burden of stroke attributable to SHS in 204 countries and regions from 1990 to 2019. We also explored whether and how the SHS-related disease burden of stroke changes with factors like age, gender, stroke subtypes, regions, and the sociodemographic index (SDI).

## Methods

### Data source

This study utilized population and disease burden data collected from the 2019 GBD study, encompassing information on strokes and their subtypes [including intracerebral hemorrhage (ICH), ischemic stroke (IS), and subarachnoid hemorrhage (SAH)] attributable to SHS exposure across different countries, regions, and genders. The 204 countries and territories analyzed were categorized into five distinct regions based on their SDI levels: high, upper-middle, lower-middle, and low SDI regions. The SDI is a comprehensive indicator calculated based on the total fertility rate among females under 25 years of age, educational attainment among those aged 15 and above, and the lagged gross domestic product *per capita*. The SDI scale ranges from 0 to 1, with 0 representing the lowest development level and 1 representing the highest.

These countries were further divided into 21 regions based on geographical locations. Data analysis employed a stratified approach, dividing age into 15 distinct groups, including 14 groups with five-year age groups (ranging from 25 to 94) and one group covering individuals aged 95 and above.

### Statistical analysis

The present study assessed the global burden of stroke attributable to SHS exposure, utilizing various metrics, including the number of deaths, age-standardized mortality rates (ASMR), and age-standardized disability-adjusted life years (DALYs) rates (ASDR), along with 95% uncertainty intervals (UI). Age-standardized rates (ASR) were calculated by standardizing for the global age structure, an essential step when comparing diverse populations across different geographic locations and periods. The estimated annual percentage changes (EAPC) were computed as 100 × (exp(β)−1), with 95% confidence intervals (CI) derived from the linear regression model. An increasing ASR trend was defined as an EAPC estimate with a lower 95% CI boundary >0, while a decreasing ASR was defined as an EAPC estimate with an upper 95% CI boundary <0. Statistical analyses were performed in R-Studio version 4.1.2, with statistical significance at *p* < 0.05. Detailed research methodology has been documented in prior publications (6, 9, 10).

## Results

### The burden of stroke attributable to SHS during 1990–2019

Globally, a total of 201.1 thousand cases of stroke deaths were attributable to SHS in 2019; the ASMR and ASDR were 2.5 (95% UI: 1.9–3.2) and 61.5 (95% UI: 46–78.8) per 100,000 population. It is observed that the number of stroke deaths is slightly higher in females, totaling 116.2 thousand The ASMR for females is 2.7 per 100,000 population, which is slightly higher than the 2.3 per 100,000 population for males. Additionally, the ASDR for females is notably higher than that for. Regarding stroke subtypes, ICH has the highest number of deaths at 101.3 thousand, followed by IS at 86.2 thousand. The ASMR and ASDR is highest for ICH. SAH has the most minor burden among stroke subtypes (Table 1).

**Table 1:** Burden of stroke attributable to secondhand smoke in 1990 and 2019, and its temporal trends from 1990 to 2019.

Characteristics	1990			2019			EAPC	
	Incident cases, *n* × 10^3^ (95% UI)	ASMR per 100,000, *n* (95% UI)	ASDR per 100,000, *n* (95% UI)	Incident cases, *n* × 10^3^ (95% UI)	ASMR per 100,000, *n* (95% UI)	ASDR per 100,000, *n* (95% UI)	ASMR	ASDR
**Global**	162.6 (118.4–206.3)	4.3 (3.2–5.4)	105.6 (78.4–134.3)	201.1 (148.8–257.6)	2.5 (1.9–3.2)	61.5 (46–78.8)	−2.08 (−2.21–1.95)	-2.08 (−2.19–1.97)
**Sex**								
Female	102.3 (74.2–130.6)	4.9 (3.6–6.2)	125.8 (92.8–159.6)	116.2 (87–149.7)	2.7 (2–3.4)	68.4 (51.2–89.1)	−2.37 (−2.52–2.23)	−2.37 (−2.51–2.24)
Male	60.3 (43.8–78)	3.5 (2.6–4.6)	83.9 (61.1–108.1)	84.9 (60.5–112)	2.3 (1.7–3)	54.1 (39–70.7)	−1.64 (−1.75–1.52)	−1.64 (−1.74–1.55)
**Subtype**								
Intracerebral hemorrhage	85.8 (62.1–110.9)	2.2 (1.6–2.8)	57.6 (41.8–74.1)	101.3 (75.1–131.4)	1.2 (0.9–1.6)	32.6 (23.8–42.1)	−2.05 (−2.27–1.84)	−2.09 (−2.27–1.91)
Ischemic stroke	56.9 (41.4–72.9)	1.6 (1.2–2.1)	33.5 (24.3–42.7)	86.2 (63.7–112.6)	1.1 (0.8–1.4)	23.5 (17.3–30)	−1.59 (−1.7–1.49)	−1.46 (−1.55–1.36)
Subarachnoid hemorrhage	19.9 (13.5–26.3)	0.5 (0.3–0.7)	14.5 (9.9–19.1)	13.5 (10–17.7)	0.2 (0.1–0.2)	5.4 (3.9–7.1)	−4.51 (−4.91–4.1)	−3.99 (−4.32–3.66)
**Socio-demographic index**								
Low SDI	6.6 (4.5–9)	2.9 (2–3.9)	75.2 (51.8–100.3)	10.7 (7.4–14)	2.1 (1.5–2.8)	54.5 (37.7–71.7)	−1.31 (−1.38–1.23)	−1.31 (−1.39–1.23)
Low-middle SDI	26.8 (19.1–35.1)	4.8 (3.5–6.3)	117.8 (85.1–153.9)	42.8 (31.2–55.1)	3.3 (2.4–4.2)	79.5 (58.9–104.1)	−1.45 (−1.53–1.36)	−1.48 (−1.55–1.4)
Middle SDI	59.7 (43.6–76.6)	6.4 (4.7–8.3)	150.2 (111–192.8)	84.5 (62.6–109.1)	3.7 (2.7–4.7)	84 (62.4–108)	−2.01 (−2.13–1.89)	−2.08 (−2.16–1.99)
High-middle SDI	53.4 (39.1–68)	5.3 (4–6.7)	123.7 (91.9–155.9)	53.1 (39.5–68.3)	2.6 (2–3.4)	61.3 (45.6–78.2)	−2.81 (−3.09–2.54)	−2.83 (−3.09–2.56)
High SDI	16 (12.1–20)	1.6 (1.2–1.9)	41.5 (31.7–52)	10 (7.6–12.7)	0.5 (0.4–0.7)	16.4 (12.3–20.6)	−4.13 (−4.34–3.91)	−3.59 (−3.79–3.39)
**Region**								
Andean Latin America	0.3 (0.2–0.3)	1.2 (0.9–1.6)	34.9 (24.8–46.6)	0.3 (0.2–0.4)	0.4 (0.3–0.6)	13 (8.6–18.2)	−3.82 (−4.16–3.49)	−3.77 (−4.07–3.46)
Australasia	0.2 (0.2–0.3)	1 (0.7–1.3)	24.3 (18.3–30.7)	0.1 (0.1–0.2)	0.3 (0.2–0.4)	8 (6–10.2)	−4.45 (−4.66–4.24)	−4.02 (−4.23–3.8)
Caribbean	0.6 (0.4–0.7)	2.3 (1.7–2.9)	61.1 (44.6–78.4)	0.7 (0.5–1)	1.4 (1–1.9)	37 (26.1–49.4)	−1.89 (−2.09–1.69)	−1.9 (−2.14–1.67)
Central Asia	2.4 (1.8–3.1)	5.5 (4–6.9)	131.6 (97.3–167)	3.2 (2.3–4.1)	4.9 (3.6–6.3)	111.2 (81.5–143.1)	−0.61 (−0.99–0.22)	−0.89 (−1.28–0.5)
Central Europe	7.6 (5.6–9.6)	5.4 (4.1–6.8)	123.5 (93.4–153.9)	5.7 (4.1–7.5)	2.6 (1.9–3.4)	55.7 (40.3–72.7)	−3.05 (−3.31–2.79)	−3.29 (−3.54–3.03)
Central Latin America	1.3 (0.9–1.6)	1.6 (1.1–2.1)	39.2 (28–50.8)	1.7 (1.1–2.3)	0.7 (0.5–1)	18.2 (12.4–25)	−3.22 (−3.45–3)	−3.11 (−3.34–2.88)
Central Sub-Saharan Africa	0.4 (0.3–0.6)	1.8 (1.2–2.5)	49.5 (32.1–70.1)	0.7 (0.4–1)	1.3 (0.8–1.8)	33.8 (21.5–48.6)	−1.34 (−1.41–1.27)	−1.42 (−1.48–1.35)
East Asia	69 (50–89.3)	9 (6.6–11.7)	201.3 (148.2–258.9)	86 (63.1–113.3)	4.5 (3.3–5.9)	97.6 (71.8–126.7)	−2.52 (−2.71–2.34)	−2.65 (−2.8–2.49)
Eastern Europe	12.9 (9.6–16.6)	4.8 (3.6–6.2)	109.5 (81–139.8)	10.2 (7.5–13)	3 (2.2–3.8)	73.7 (54.1–92.7)	−2.53 (−3.12–1.94)	−2.19 (−2.79–1.58)
Eastern Sub-Saharan Africa	1.7 (1.2–2.4)	2.3 (1.5–3.1)	63.1 (43.4–85.9)	2.6 (1.7–3.6)	1.6 (1–2.2)	41.6 (27.4–57.7)	−1.42 (−1.53–1.32)	−1.63 (−1.74–1.51)
High-income Asia Pacific	4.6 (3.5–5.8)	2.4 (1.8–3)	61.2 (46.1–76.9)	2.6 (1.9–3.3)	0.6 (0.4–0.7)	17.8 (13.3–23)	−5.42 (−5.63–5.22)	−4.57 (−4.74–4.4)
High-income North America	3 (2.3–3.7)	0.9 (0.7–1.1)	26.3 (20.4–32.8)	2.4 (1.8–3.1)	0.4 (0.3–0.5)	12.7 (9.5–16.2)	−3.31 (−3.6–3.03)	−2.96 (−3.2–2.72)
North Africa and Middle East	7.2 (5.2–9.2)	4.6 (3.3–5.9)	111.9 (82.2–142.9)	11.9 (8.8–15.2)	3 (2.2–3.8)	71.9 (53.5–91.9)	−1.52 (−1.56–1.48)	−1.54 (−1.57–1.51)
Oceania	0.2 (0.1–0.2)	6 (4–8.3)	162.4 (108.5–223.6)	0.4 (0.2–0.6)	5.5 (3.6–7.8)	153.7 (99.2–221.8)	−0.25 (−0.31–0.19)	−0.13 (−0.2–0.06)
South Asia	19.4 (13.4–26.1)	3.9 (2.7–5.2)	90.1 (63.5–119.5)	33.1 (23.5–43.6)	2.5 (1.8–3.3)	60.4 (43–80.2)	−1.81 (−1.95−1.68)	-1.6 (−1.71–1.49)
Southeast Asia	14.3 (10.1–18.5)	6 (4.3–7.7)	149.4 (108–192.2)	26.9 (19.5–35.1)	4.7 (3.4–6.1)	115.8 (83.7–151.2)	−0.67 (−0.77–0.57)	−0.74 (−0.83–0.65)
Southern Latin America	1.7 (1.2–2.1)	3.7 (2.8–4.7)	90.8 (68–114.8)	1.1 (0.8–1.5)	1.3 (1–1.7)	32.2 (23.5–40.9)	−3.83 (−4.06–3.6)	−3.99 (−4.22–3.76)
Southern Sub-Saharan Africa	0.9 (0.6–1.1)	3.2 (2.3–4.2)	83.5 (60.4–107.3)	1.1 (0.7–1.4)	2 (1.5–2.7)	47.7 (33.7–62.9)	−1.57 (−1.96–1.17)	−1.94 (−2.31–1.57)
Tropical Latin America	4.5 (3.3–5.6)	4.9 (3.7–6.2)	130.6 (97.6–165.2)	3.3 (2.4–4.2)	1.4 (1–1.8)	35.6 (26.5–45.7)	−4.59 (−4.78–4.4)	−4.73 (−4.92–4.53)
Western Europe	8.7 (6.5–11)	1.5 (1.2–1.9)	37 (28.1–45.9)	4.2 (3.1–5.4)	0.4 (0.3–0.6)	11.5 (8.8–14.4)	−4.65 (−4.85–4.45)	−4.38 (−4.56–4.19)
Western Sub-Saharan Africa	1.8 (1.2–2.5)	2.1 (1.4–2.9)	54.5 (36–76.3)	2.9 (2.1–3.9)	1.6 (1.1–2.1)	40.7 (28.5–54.7)	−1.06 (−1.15–0.97)	−1.11 (−1.2–1.01)

ASMR, age-standardized mortality rate; ASDR, age-standardized disability-adjusted life year rate; EAPC, estimated annual percentage change.

**Figure 1:**
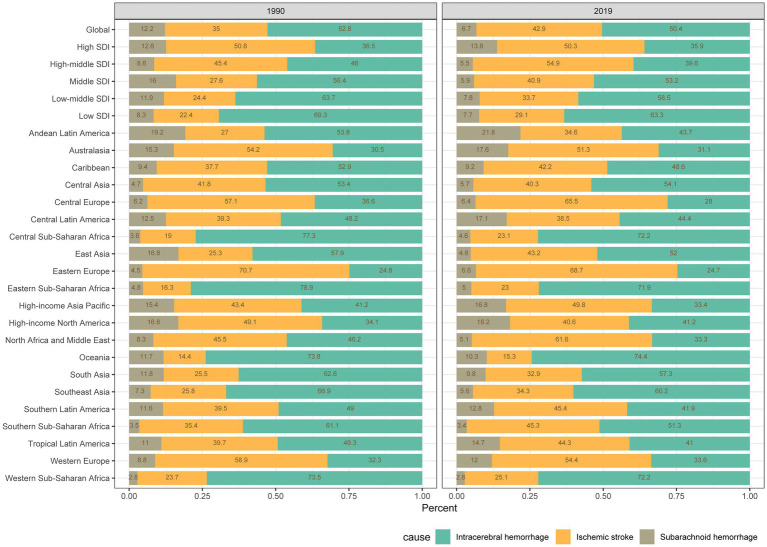
Contribution of secondhand smoke to the absolute number of deaths from intracerebral hemorrhage, ischemic stroke, and subarachnoid hemorrhage globally and in various regions from 1990 to 2019.

Disparities are evident in the burden of stroke attributable to SHS across regions with varying SDI levels. In 1990, high SDI regions recorded 16,000 stroke deaths (95% UI: 12,100–20,000), an ASMR of 1.6 per 100,000 individuals (95% UI: 1.2–1.9), and an ASDR rate of 41.5 per 100,000 (95% UI: 31.7–52). By 2019, these numbers had significantly decreased to 10,000 deaths (95% UI: 7,600–12,700), an ASMR of 0.5 per 100,000 (95% UI: 0.4–0.7), and an ASDR of 16.4 per 100,000 (95% UI: 12.3–20.6). In high and high-middle SDI regions, IS accounts for most stroke deaths (50.3 and 54.9%, respectively). However, in the middle, low-middle, and low SDI regions, ICH represents the most significant proportion of stroke mortality (53.2, 58.5, and 63.3%, respectively) (Figure 1).

**Figure 2:**
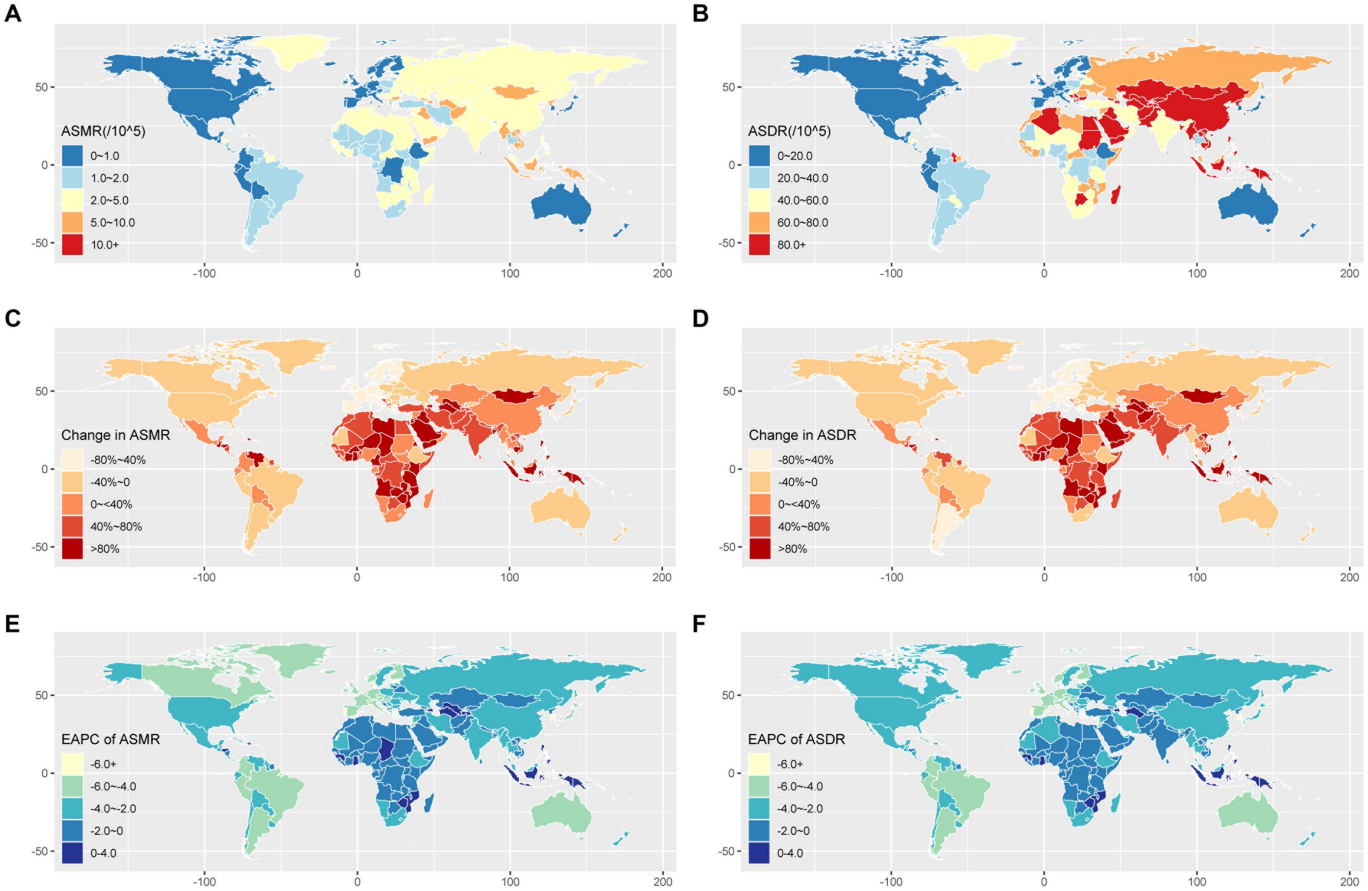
Geographical variation in stroke burden attributed to secondhand smoke across 204 countries and territories. **(A)** The ASMR of stroke burden attributable to secondhand smoke in 2019. **(B)** The ASDR of stroke burden attributable to secondhand smoke in 2019. **(C)** The percentage change in stroke ASMR from 1990 to 2019. **(D)** The percentage change in stroke ASDR from 1990 to 2019. **(E)** The EAPC of stroke ASMR from 1990 to 2019. **(F)** The EAPC of stroke ASDR from 1990 to 2019. ASDR, age-standardized disability-adjusted life-year rate; ASMR, age-standardized mortality rate; EAPC, estimated annual percentage change.

From a regional perspective in 2019, Eastern Asia had the highest death of stroke contributable to SHS at 86 thousand cases, followed by South Asia and Southeast Asia (Table 1). The regions with the highest ASMR were Oceania, and Southeast Asia. These regions also exhibited elevated ASDR (Table 1 and Figures 2A,B). In contrast, Australasia had the lowest number of stroke deaths at 0.1 × 10^3^ (95% UI: 0.1 × 10^3^–0.2 × 10^3^) cases, ASMR of 0.3 (95% UI: 0.2–0.4) per 100,000, and ASDR of 8 (95% UI: 6–10.2) per 100,000. In 2019, China, India and Indonesia had the highest number of stroke deaths attributable to SHS exposure globally, with 83.3, 23.4 and 13.1 thousand deaths, respectively (Supplementary Table S3).

### Global burden of stroke mortality attributable to SHS by age and sex

**Figure 3:**
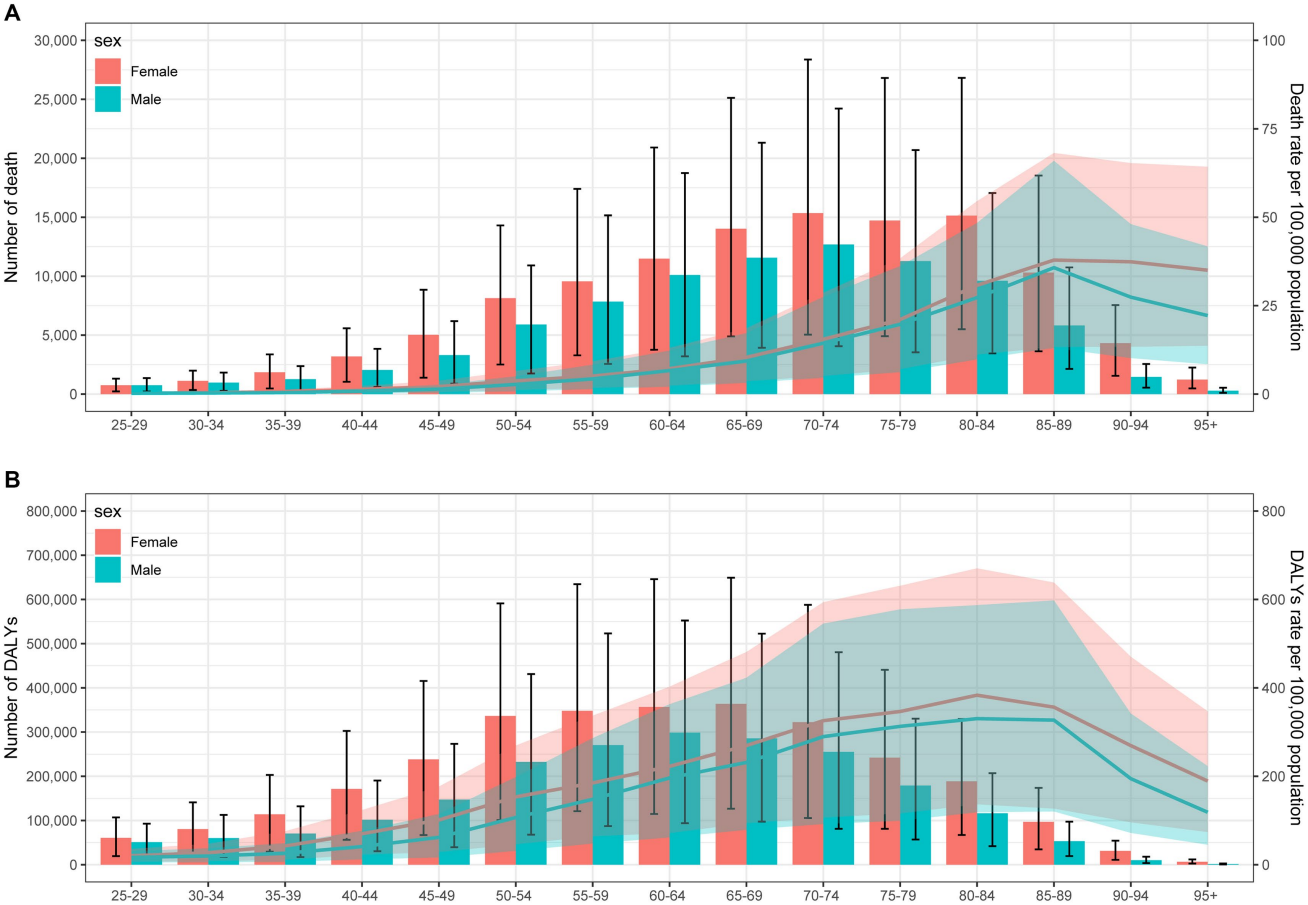
Age-specific mortality **(A)** and DALYs **(B)** of stroke attributable to secondhand smoke by gender in 2019. DALY, disability-adjusted life years.

Globally, the burden of stroke attributable to SHS was predominantly concentrated among the elderly, with women experiencing a higher burden than men (Figure 3). As shown in Figure 3A, the number of stroke deaths and mortality rates attributable to SHS in 2019 increase with age for both genders, with deaths peaking at ages 70–74 and mortality rates peaking at ages 85–89. Across all age groups, women had more deaths and higher mortality rates than men. Figure 3B presents the number and rate of DALYs due to stroke attributable to SHS in 2019. Similarly, the results indicate that the number and rate of DALYs increase with age, with the number and rates of DALYs and DALY peaking at ages 60–69 and 80–89, respectively. The burden of stroke subtypes, including ICH, IS, and SAH, attributable to SHS by age and sex are presented in Supplementary Figures S1–S3.

### Relationship between SDI and SHS–related stroke burden

**Figure 4:**
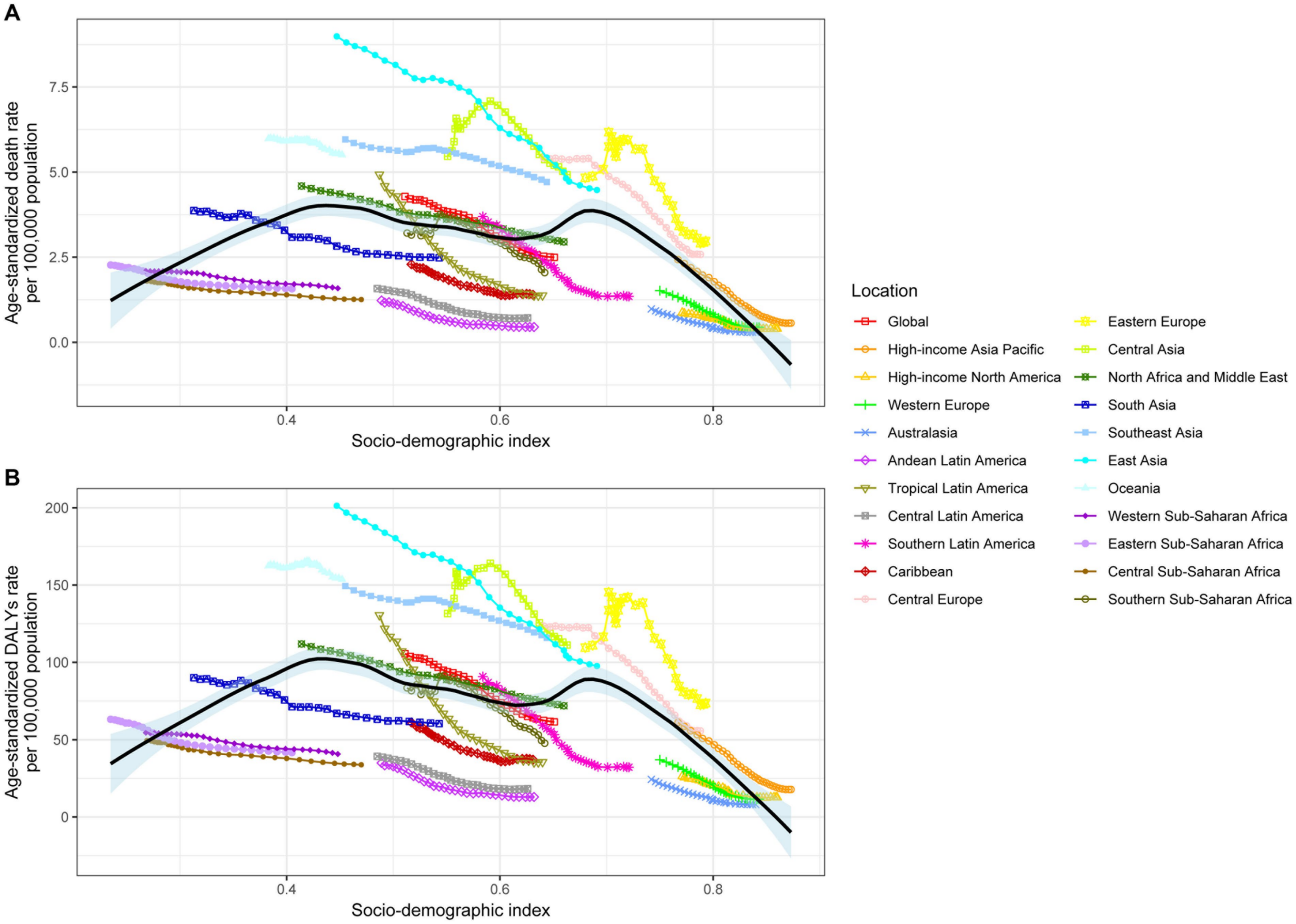
Age-standardized stroke death rates **(A)** and DALYs **(B)** attributed to secondhand smoke by SDI, 1990–2019. DALY, disability-adjusted life years.

**Figure 5:**
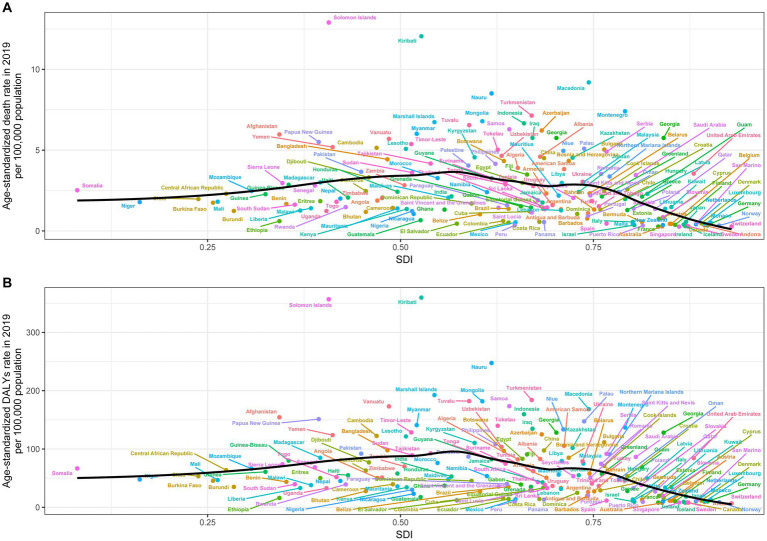
Age-standardized rates of stroke death **(A)** and DALYs **(B)** attributable to secondhand smoke in 204 countries by SDI in 2019. DALY, disability-adjusted life years.

Figure 4 shows the relationship between ASMR attributable to SHS and SDI across 21 GBD regions globally from 1990 to 2019. The results demonstrate an “M” shaped nonlinear relationship between ASMR and SDI, with ASMR gradually increasing when SDI < 0.45 but rapidly decreasing when SDI > 0.7. A similar relationship was observed between ASDR and SDI. In 2019, across 204 countries and territories globally, the relationships between ASMR and ASDR attributable to SHS and SDI show an initial increase followed by a decrease in ASMR and ASDR as SDI rises (Figure 5).

### Temporal trends in SHS-attributable stroke burden from 1990 to 2019

Compared to 1990, the number of stroke deaths attributable to SHS increased in 2019. However, the ASMR and ASDR decreased, with EAPC rates of −2.08% (95% CI: −2.21% to −1.95%) and − 2.08% (95% CI: −2.19% to −1.97%) for ASMR and ASDR, respectively. When considering gender differences, females exhibit a more significant reduction in EAPC for both ASMR and ASDR compared to males. Regarding stroke subtypes, SAH demonstrates the most significant decline in EAPC for both ASMR and ASDR, reaching −4.51% (95% CI: −4.91% to −4.1%) and − 3.99% (95% CI: −4.32% to −3.66%), and ICH follows, with EAPC rates of −2.05% for ASMR and − 2.09% for ASDR. IS has an EAPC of −1.59% for ASMR and − 1.46% for ASDR (Table 1). For ICH and IS, the number of stroke deaths in 2019 increased compared to 1990 (Supplementary Tables S1, S2). However, the EAPC results for both ASMR and ASDR indicated a decrease over this period. For SAH, the absolute number of deaths decreased, aligned with the declining trends for ASMR and ASDR.

It is noteworthy that high SDI regions displayed the most accelerated rate of decline, exhibiting an EAPC of −4.13% (95% CI: −4.34% to −3.91%) for ASMR and − 3.59% (95% CI: −3.79% to −3.39%) for ASDR. In contrast, regions with low SDI experienced a relatively more gradual reduction across these metrics, with an ASMR EAPC of −1.31% (95% CI: −1.38% to −1.23%) and an ASDR EAPC of −1.31% (95% CI: −1.39% to −1.23%). In high and high-middle SDI regions, we observed a decline in the number of deaths, ASMR, and ASDR from stroke. In contrast, the other 3 SDI regions displayed a reduction in ASMR and ASDR but did not exhibit a concurrent decrease in deaths (Table 1 and Supplementary Figure S4). The most rapid reductions in ASMR and ASDR for ICH and IS are still observed in the High SDI regions. As for SAH, the most rapid reductions in ASMR and ASDR are found in the Middle SDI regions [with EAPC values of −6.43% (95% CI: −7.09% to −5.77%) and −5.64% (95% CI: −6.21% to −5.07%), respectively] (Supplementary Tables S1–S3 and Supplementary Figures S5–S7).

Regionally in 2019, high-income Asia Pacific and Western Europe exhibited the most significant decreases in ASMR, with EAPC values of −5.42% and − 4.65%, respectively. For ASDR, Tropical Latin America and High-income Asia Pacific had the most substantial reductions in EAPC (Table 1 and Figures 2C–F). The variations in ASMR and ASDR for the subtypes (ICH, IS, and SAH) across different regions from 1990 to 2019 are available in Supplementary Tables S1–S3.

## Discussion

This study leveraged data from the GBD research program to evaluate the global burden of stroke attributable to SHS exposure across 204 countries and territories from 1990 to 2019. The results revealed that SHS continues to impose a substantial stroke burden globally. In 2019 alone, approximately 2.01 million stroke deaths were attributable to SHS, with an ASMR of 2.5 per 100,000 and an ASDR of 61.5 per 100,000. Compared to 1990, the total number of stroke deaths rose, yet the ASRs declined, with EAPC reductions of 2.08% for both ASMR and ASDR. Significant disparities emerged across countries and regions, with a greater burden among females versus males and the elderly carrying the highest burden. ICH and IS constituted the major components of the stroke burden attributed to SHS. High-income regions demonstrated the most remarkable reductions, while low-income regions lagged in curbing disease burden.

SHS, also known as environmental tobacco smoke, primarily comprises sidestream smoke released from the burning cigarette tip (~85%) in addition to exhaled mainstream smoke from smokers (~15%) (11). Indoors, SHS can persist for hours, with toxicity increasing over time (12). SHS contains over 7,000 chemicals, hundreds of which are hazardous and several carcinogenic (13). Exposure is associated with adverse health effects including sudden infant death syndrome, ear and respiratory infections in children, asthma attacks, coronary heart disease, and lung cancer among nonsmokers (14). Current evidence suggests potential biological mechanisms linking SHS exposure to the pathogenesis of both ischemic and hemorrhagic stroke. At the molecular level, reactive oxidative species in SHS may directly impair vascular endothelial cell function (15), disrupting the blood–brain barrier—a key mechanism underlying IS (16). SHS may also promote coagulation and platelet activation to form arterial blood clots, leading to cerebral vessel occlusion and IS (17, 18). Regarding hemorrhagic stroke, SHS can stimulate cerebral vascular smooth muscle cell proliferation and extracellular matrix protein expression, contributing to aneurysm formation and rupture (19, 20). Additionally, carbon monoxide in SHS binds to hemoglobin, forming carboxyhemoglobin and reducing oxygen-carrying capacity, which has also been implicated in hemorrhagic stroke (21). SHS may further participate in developing both stroke subtypes by modulating various channel activities and gene expression, such as activating calcium-dependent potassium channels and upregulating matrix metalloproteinase expression (e.g., MMP-2 and MMP-9) (22–24).

This findings from this study are comparable to previous research on similar topics. Okekunle et al. evaluated the association of SHS with stroke among indigenous Africans, finding SHS was positively associated with stroke (25). A prior case–control study conducted in China revealed a heightened mortality risk from various stroke subtypes related to SHS exposure, including a 10% increased risk for hemorrhagic stroke and a 12% increased risk for IS (26). These prior findings reinforce the conclusions from our study identifying SHS as a risk factor for stroke. Unlike this previous research, our study leveraged the GBD 2019 dataset to evaluate 204 countries, utilized standardized metrics to quantify disease burden, and provided granular analyses across diverse population groups, thereby furnishing more comprehensive evidence linking SHS and stroke. Our detailed findings demonstrating the considerable stroke burden across countries, genders, and subtypes underscore the imperative for governments globally to implement proactive interventions aimed at reducing SHS exposure and promoting smoke-free environments.

This study reveals the burden of stroke related to SHS exposure is highest among those aged 65 years and older. Importantly, the elderly already represent a high-risk population for stroke due to age-related physiological decline and atherosclerosis. Within this vulnerable elderly population, exposure to SHS further increases the stroke risk (27). Prior research confirms SHS exposure is prevalent among older adults, contributing to approximately 41,000 deaths yearly in American adults. Due to age-related decreases in physiological function and higher rates of comorbidities, older individuals remain especially susceptible to tobacco’s harmful effects (28, 29). These findings underscore the importance of reducing SHS exposure among the elderly. Additionally, a major finding from this study highlights across all age groups, females faced a considerably higher burden of stroke attributable to SHS than males. Several factors may explain this gender disparity, including smaller arterial diameters and other specific cerebrovascular characteristics in females that could render them more susceptible to SHS’s toxic impacts (30). Compared to men, females also have greater cumulative exposure due to their longer average lifespan (31). Postmenopausal women further lack estrogen’s protective effects, conferring greater vulnerability to secondhand smoke (31). In summary, irrespective of the age, females consistently exhibit a higher stroke burden, underscoring their heightened sensitivity to SHS’s hazards. Prolonged exposure may prompt continuous escalation of this burden. These findings underscore the urgent need to develop specialized interventions focused on preventing SHS exposure among women in particular.

From 1990 to 2019, decreases emerged in both ASMR and the ASDR, with an EAPC of approximately −2.08% for each. This decline may be attributed to implementing policies and legislation prohibiting smoking in public places, such as public transportation, restaurants, and hospitals, as well as workplaces associated with the hospitality industry (32, 33). However, despite enacting smoking bans, compliance levels vary significantly across countries due to differing political and economic factors, especially in low- and middle-income nations (34, 35). Previous research indicates that among certain populations, lower socioeconomic status associates with higher exposure to SHS (36, 37). Aligning with those findings, disparities emerged in the SHS-attributable stroke burden between regions with varying SDI levels in this study. Specifically, high SDI regions demonstrated the most pronounced reductions across indicators, likely reflecting more comprehensive tobacco control policies, increased public health awareness, and superior healthcare infrastructure. In contrast, low SDI regions exhibited relatively slower control of disease burden (38). These findings highlight the potential benefits of elevating socioeconomic development for reducing the burden of stroke attributed to SHS. However, irrespective of regional developmental levels, implementing comprehensive interventions, including robust tobacco control policies and widespread public education, could more effectively facilitate these improvements.

By leveraging the latest GBD 2019 dataset, this study comprehensively evaluated the global burden of stroke attributable to SHS exposure over 30 years spanning 204 countries and regions. The findings provide critical insights to guide the development of targeted SHS prevention and control strategies at both the global and regional levels. This research also establishes an evidence-based foundation for formulating stroke prevention and control policies tailored to specific countries, genders, and age groups. Moreover, these results underscore the persistent need for ongoing efforts to curb SHS exposure and enhance public awareness of the associated health hazards. However, this study has some limitations. First, omitting asymptomatic stroke cases may have led to underestimating the total disease burden of SHS-induced strokes. Second, dependence on GBD model estimates rather than actual data, especially for low- and middle-income nations, may have impacted results. Third, variability in data quality between regions may affect comparability. Finally, more granular age stratification and accounting for interaction with other stroke risk factors could have improved accuracy.

In summary, this study demonstrates that SHS substantially contributes to the global burden of stroke, with salient variations across populations and regions. Future research may benefit from more robust cohort studies to elucidate interactions and mechanisms. Ongoing research should focus on lower-income countries to achieve reductions in the stroke burden globally.

## Data availability statement

The original contributions presented in the study are included in the article/Supplementary material, further inquiries can be directed to the corresponding author.

## Author contributions

XY: Conceptualization, Data curation, Investigation, Methodology, Software, Writing – original draft, Writing – review & editing. JS: Conceptualization, Methodology, Visualization, Writing – review & editing. WZ: Conceptualization, Writing – review & editing.
