# Supplementary material for: Global burden of stroke attributable to secondhand smoke in 204 countries and territories from 1990 to 2019: analysis of the global burden of disease study
Source: Front Neurol. 2024 Jan 26;15:1320033. doi: 10.3389/fneur.2024.1320033 (PMC10853451; doi:10.3389/fneur.2024.1320033)
Supplement: Supplementary file 1 [file Data_Sheet_1.docx]

# Supplementary Figure legends

Supplementary Figure 1: Age-Specific Mortality (A) and DALYs(B) of ICH Attributable to Secondhand smoke by Gender in 2019.
DALY: disability-adjusted life years; ICH: intracerebral hemorrhage.

Supplementary Figure 2: Age-Specific Mortality (A) and DALYs(B) of IS Attributable to Secondhand smoke by Gender in 2019.
DALY: disability-adjusted life years; IS: ischemic stroke.

Supplementary Figure 3: Age-Specific Mortality (A) and DALYs(B) of SAH Attributable to Secondhand smoke by Gender in 2019.
DALY: disability-adjusted life years; SAH: subarachnoid hemorrhage.

Supplementary Figure 4: Temporal Trends in ASMR (per 100,000 Population) (A) and ASDR (per 100,000 Population) (B) for Stroke Attributed to Secondhand Smoke in Global and SDI Regions, 1990–2019.
ASMR: age-standardized mortality rates, ASDR: age-standardized disability-adjusted life years rates.

Supplementary Figure 5: Temporal Trends in ASMR (per 100,000 Population) (A) and ASDR (per 100,000 Population) (B) for ICH Attributed to Secondhand Smoke in Global and SDI Regions, 1990–2019.
ASMR: age-standardized mortality rates, ASDR: age-standardized disability-adjusted life years rates; ICH: intracerebral hemorrhage.

Supplementary Figure 6: Temporal Trends in ASMR (per 100,000 Population) (A) and ASDR (per 100,000 Population) (B) for IS Attributed to Secondhand Smoke in Global and SDI Regions, 1990–2019.
ASMR: age-standardized mortality rates, ASDR: age-standardized disability-adjusted life years rates; IS: ischemic stroke.

Supplementary Figure 7: Temporal Trends in ASMR (per 100,000 Population) (A) and ASDR (per 100,000 Population) (B) for SAH Attributed to Secondhand Smoke in Global and SDI Regions, 1990–2019.
ASMR: age-standardized mortality rates, ASDR: age-standardized disability-adjusted life years rates; SAH: subarachnoid hemorrhage.

# Supplementary Tables

**Supplementary Table 1. ICH burden attributable to ambient secondhand smoke in 1990 and 2019, and its temporal trends from 1990 to 2019.**

| **Characteristics** | **1990** | | |  | **2019** | | |  | **EAPC** | |
| --- | --- | --- | --- | --- | --- | --- | --- | --- | --- | --- |
|  | **Incident cases,**  **n × 10^3^ (95% UI)** | **ASMR per 100,000,**  **n (95% UI)** | **ASDR per 100,000,**  **n (95% UI)** |  | **Incident cases,**  **n × 10^3^ (95% UI)** | **ASMR per 100,000,**  **n (95% UI)** | **ASDR per 100,000,**  **n (95% UI)** |  | **ASMR** | **ASDR** |
| **Global** | 85.8 (62.1-110.9) | 2.2 (1.6-2.8) | 57.6 (41.8-74.1) |  | 101.3 (75.1-131.4) | 1.2 (0.9-1.6) | 32.6 (23.8-42.1) |  | -2.05 (-2.27--1.84) | -2.09 (-2.27--1.91) |
| **Sex** |  |  |  |  |  |  |  |  |  |  |
| Female | 53.2 (37.7-69.1) | 2.5 (1.8-3.2) | 68.6 (49.3-88.3) |  | 58 (43.1-75) | 1.3 (1-1.7) | 36 (26.5-46.9) |  | -2.33 (-2.55--2.11) | -2.41 (-2.6--2.22) |
| Male | 32.6 (23.4-42.5) | 1.8 (1.3-2.3) | 46.2 (33.1-59.7) |  | 43.4 (30.9-57.6) | 1.1 (0.8-1.5) | 29.1 (20.9-37.9) |  | -1.66 (-1.88--1.44) | -1.65 (-1.83--1.47) |
| **Socio-demographic index** |  |  |  |  |  |  |  |  |  |  |
| Low SDI | 4.6 (3.1-6.3) | 1.9 (1.3-2.6) | 51.9 (35-70.5) |  | 6.8 (4.6-9) | 1.2 (0.9-1.7) | 34.6 (23.6-46.2) |  | -1.64 (-1.73--1.55) | -1.61 (-1.7--1.52) |
| Low-middle SDI | 17.1 (12-22.7) | 2.9 (2-3.8) | 74.9 (53.1-98.3) |  | 25 (17.9-33) | 1.8 (1.3-2.4) | 47.6 (34-63.2) |  | -1.61 (-1.72--1.5) | -1.63 (-1.71--1.54) |
| Middle SDI | 33.6 (24.5-43.7) | 3.4 (2.5-4.5) | 84.5 (61.4-108.9) |  | 44.9 (33.4-58.2) | 1.9 (1.4-2.4) | 45.1 (33.4-58.3) |  | -2.07 (-2.35--1.79) | -2.13 (-2.35--1.91) |
| High-middle SDI | 24.6 (17.9-31.5) | 2.3 (1.7-3) | 60.1 (43.9-76.6) |  | 21 (15.7-27.1) | 1 (0.8-1.3) | 26.7 (19.8-34.3) |  | -3.15 (-3.52--2.77) | -3.16 (-3.52--2.8) |
| High SDI | 5.8 (4.4-7.3) | 0.6 (0.4-0.7) | 17.2 (12.8-21.7) |  | 3.6 (2.7-4.6) | 0.2 (0.2-0.3) | 6.5 (4.8-8.2) |  | -3.92 (-4.11--3.72) | -3.73 (-3.93--3.53) |
| **Region** |  |  |  |  |  |  |  |  |  |  |
| Andean Latin America | 0.1 (0.1-0.2) | 0.6 (0.4-0.9) | 19.3 (13.1-26.4) |  | 0.1 (0.1-0.2) | 0.2 (0.1-0.3) | 6 (3.8-8.6) |  | -4.53 (-4.9--4.16) | -4.45 (-4.8--4.11) |
| Australasia | 0.1 (0-0.1) | 0.3 (0.2-0.4) | 8.1 (5.8-10.3) |  | 0 (0-0.1) | 0.1 (0.1-0.1) | 2.6 (1.9-3.3) |  | -4.05 (-4.22--3.87) | -4.06 (-4.27--3.86) |
| Caribbean | 0.3 (0.2-0.4) | 1.2 (0.8-1.5) | 35.2 (25.2-46.4) |  | 0.4 (0.2-0.5) | 0.7 (0.5-0.9) | 20.2 (13.8-27.6) |  | -2.14 (-2.38--1.91) | -2.13 (-2.4--1.86) |
| Central Asia | 1.3 (0.9-1.7) | 2.8 (2-3.6) | 73.2 (53.1-94.6) |  | 1.7 (1.3-2.3) | 2.4 (1.8-3.1) | 60 (43.6-77.5) |  | -0.73 (-1.23--0.24) | -1.04 (-1.5--0.56) |
| Central Europe | 2.8 (2.1-3.5) | 1.9 (1.4-2.4) | 50.5 (37.6-63.9) |  | 1.6 (1.1-2.1) | 0.8 (0.5-1) | 19 (13.3-25) |  | -3.96 (-4.31--3.61) | -4.2 (-4.56--3.84) |
| Central Latin America | 0.6 (0.4-0.8) | 0.7 (0.5-0.9) | 19.6 (13.8-25.8) |  | 0.7 (0.5-1) | 0.3 (0.2-0.4) | 8.6 (5.7-11.9) |  | -3.54 (-3.79--3.3) | -3.49 (-3.74--3.24) |
| Central Sub-Saharan Africa | 0.3 (0.2-0.5) | 1.3 (0.8-1.8) | 37.6 (24.4-53.8) |  | 0.5 (0.3-0.7) | 0.8 (0.5-1.2) | 23.9 (15.2-34.8) |  | -1.63 (-1.71--1.55) | -1.66 (-1.74--1.58) |
| East Asia | 39.9 (28.7-52.8) | 5.1 (3.7-6.7) | 115.3 (83.3-149.7) |  | 44.8 (32.7-59.2) | 2.3 (1.7-3) | 50.9 (37.4-66.6) |  | -2.75 (-3.13--2.37) | -2.82 (-3.15--2.49) |
| Eastern Europe | 3.2 (2.2-4.2) | 1.1 (0.8-1.5) | 32.3 (23.1-42.1) |  | 2.5 (1.8-3.2) | 0.8 (0.6-1) | 24.1 (17.3-31.3) |  | -2.28 (-2.98--1.57) | -1.92 (-2.66--1.18) |
| Eastern Sub-Saharan Africa | 1.4 (0.9-1.9) | 1.7 (1.1-2.3) | 49.3 (33.4-67.7) |  | 1.9 (1.2-2.6) | 1 (0.7-1.5) | 29.6 (19.1-41.8) |  | -1.85 (-1.97--1.73) | -1.98 (-2.11--1.86) |
| High-income Asia Pacific | 1.9 (1.4-2.4) | 1 (0.7-1.2) | 27 (20.1-34.3) |  | 0.9 (0.6-1.1) | 0.2 (0.2-0.3) | 6.5 (4.8-8.5) |  | -5.38 (-5.57--5.18) | -5.11 (-5.27--4.94) |
| High-income North America | 1 (0.8-1.3) | 0.3 (0.2-0.4) | 9.7 (7.4-12.2) |  | 1 (0.7-1.3) | 0.2 (0.1-0.2) | 5.2 (3.9-6.8) |  | -2.61 (-2.86--2.36) | -2.65 (-2.89--2.42) |
| North Africa and Middle East | 3.3 (2.4-4.4) | 1.9 (1.4-2.5) | 52.5 (38.2-68.5) |  | 4 (2.9-5.1) | 0.9 (0.6-1.1) | 24.9 (18.1-32.3) |  | -2.9 (-2.99--2.8) | -2.76 (-2.84--2.67) |
| Oceania | 0.1 (0.1-0.2) | 4.2 (2.7-5.9) | 116.3 (75.5-163.5) |  | 0.3 (0.2-0.4) | 3.9 (2.5-5.6) | 110.9 (71-163.9) |  | -0.21 (-0.29--0.14) | -0.09 (-0.17-0) |
| South Asia | 12.2 (8.2-16.8) | 2.2 (1.5-3.1) | 55.8 (38-76.4) |  | 19 (12.9-26) | 1.3 (0.9-1.8) | 35.6 (24.5-48.3) |  | -1.97 (-2.09--1.85) | -1.73 (-1.84--1.62) |
| Southeast Asia | 9.6 (6.7-12.6) | 3.7 (2.6-4.9) | 99.9 (71.9-131) |  | 16.2 (11.4-21.7) | 2.6 (1.9-3.5) | 71 (50-94.8) |  | -1.05 (-1.18--0.93) | -1.04 (-1.15--0.92) |
| Southern Latin America | 0.8 (0.6-1) | 1.8 (1.3-2.3) | 48.8 (35.4-62.8) |  | 0.5 (0.3-0.6) | 0.6 (0.4-0.7) | 15.4 (11-20) |  | -4.32 (-4.57--4.07) | -4.5 (-4.75--4.24) |
| Southern Sub-Saharan Africa | 0.5 (0.4-0.7) | 1.8 (1.3-2.3) | 53.2 (38.2-68.7) |  | 0.5 (0.4-0.7) | 0.9 (0.6-1.2) | 25.6 (17.6-34.1) |  | -2.29 (-2.73--1.86) | -2.57 (-2.99--2.16) |
| Tropical Latin America | 2.2 (1.6-2.8) | 2.2 (1.6-2.8) | 67.7 (49.4-87.4) |  | 1.4 (1-1.8) | 0.5 (0.4-0.7) | 16.2 (11.8-21.3) |  | -5.14 (-5.33--4.95) | -5.26 (-5.46--5.05) |
| Western Europe | 2.8 (2.1-3.6) | 0.5 (0.4-0.6) | 14.7 (10.9-18.4) |  | 1.4 (1-1.8) | 0.2 (0.1-0.2) | 4.4 (3.3-5.6) |  | -4.33 (-4.49--4.17) | -4.54 (-4.71--4.37) |
| Western Sub-Saharan Africa | 1.3 (0.9-1.9) | 1.5 (1-2.1) | 40 (26.4-56.3) |  | 2.1 (1.5-2.9) | 1.1 (0.7-1.4) | 29 (20-39.9) |  | -1.18 (-1.27--1.08) | -1.2 (-1.29--1.1) |

ASMR: age-standardized mortality rate; ASDR: age-standardized disability-adjusted life year rate; EAPC: estimated annual percentage change.

**Supplementary Table 2. IS burden attributable to secondhand smoke in 1990 and 2019, and its temporal trends from 1990 to 2019.**

| **Characteristics** | **1990** | | | |  | **2019** | | |  | **EAPC** | |
| --- | --- | --- | --- | --- | --- | --- | --- | --- | --- | --- | --- |
|  | **Incident cases,**  **n × 10^3^ (95% UI)** | **ASMR per 100,000,**  **n (95% UI)** | **ASDR per 100,000,**  **n (95% UI)** | |  | **Incident cases,**  **n × 10^3^ (95% UI)** | **ASMR per 100,000,**  **n (95% UI)** | **ASDR per 100,000,**  **n (95% UI)** |  | **ASMR** | **ASDR** |
| **Global** | 56.9 (41.4-72.9) | 1.6 (1.2-2.1) | 33.5 (24.3-42.7) | |  | 86.2 (63.7-112.6) | 1.1 (0.8-1.4) | 23.5 (17.3-30) |  | -1.59 (-1.7--1.49) | -1.46 (-1.55--1.36) |
| **Sex** |  |  |  | |  |  |  |  |  |  |  |
| Female | 35.8 (26.3-46.3) | 1.8 (1.3-2.3) | 38.2 (27.7-48.3) | |  | 50 (37-65.6) | 1.1 (0.8-1.5) | 25.8 (19.2-33) |  | -1.84 (-1.98--1.7) | -1.65 (-1.77--1.52) |
| Male | 21.1 (15.2-27.7) | 1.4 (1-1.8) | 27.9 (20.1-36.4) | |  | 36.2 (25.6-47.8) | 1 (0.7-1.4) | 20.8 (14.7-27.2) |  | -1.19 (-1.25--1.12) | -1.14 (-1.2--1.08) |
| **Socio-demographic index** |  |  |  |  |  |  |  |  |  |  |  |
| Low SDI | 1.5 (0.9-2.2) | 0.8 (0.5-1.2) | 16.8 (11-24.5) | |  | 3.1 (2.1-4.3) | 0.7 (0.5-1) | 15.4 (10.6-20.9) |  | -0.54 (-0.6--0.48) | -0.4 (-0.45--0.35) |
| Low-middle SDI | 6.5 (4.5-9) | 1.4 (1-2) | 28.2 (19.7-38.2) | |  | 14.4 (10.2-19) | 1.2 (0.9-1.6) | 24.6 (17.9-31.8) |  | -0.68 (-0.77--0.59) | -0.58 (-0.64--0.52) |
| Middle SDI | 16.5 (11.6-21.7) | 2 (1.5-2.7) | 41.4 (29.9-53.3) | |  | 34.6 (25.2-45.4) | 1.6 (1.2-2.1) | 32.9 (24.2-42.1) |  | -0.83 (-0.9--0.75) | -0.82 (-0.87--0.76) |
| High-middle SDI | 24.3 (17.9-31.1) | 2.5 (1.9-3.3) | 50.6 (37-64.1) | |  | 29.1 (21.5-37.8) | 1.4 (1.1-1.9) | 29.6 (21.8-38.1) |  | -2.35 (-2.58--2.11) | -2.26 (-2.49--2.03) |
| High SDI | 8.1 (6-10.4) | 0.8 (0.6-1) | 16.3 (12.1-20.4) | |  | 5 (3.7-6.5) | 0.2 (0.2-0.3) | 6.4 (4.8-8.1) |  | -4.56 (-4.82--4.31) | -3.68 (-3.92--3.44) |
| **Region** |  |  |  | |  |  |  |  |  |  |  |
| Andean Latin America | 0.1 (0-0.1) | 0.4 (0.3-0.5) | 8.1 (5.5-10.9) | |  | 0.1 (0.1-0.1) | 0.2 (0.1-0.2) | 3.5 (2.3-4.9) |  | -3.38 (-3.71--3.04) | -3.31 (-3.64--2.97) |
| Australasia | 0.1 (0.1-0.2) | 0.5 (0.4-0.7) | 10.4 (7.6-13.4) | |  | 0.1 (0.1-0.1) | 0.1 (0.1-0.2) | 3 (2.2-3.8) |  | -5.21 (-5.48--4.94) | -4.63 (-4.93--4.33) |
| Caribbean | 0.2 (0.2-0.3) | 0.9 (0.7-1.2) | 18.6 (13.5-23.8) | |  | 0.3 (0.2-0.4) | 0.6 (0.4-0.8) | 12.1 (8.6-16.2) |  | -1.62 (-1.77--1.48) | -1.57 (-1.73--1.4) |
| Central Asia | 1 (0.7-1.3) | 2.4 (1.8-3.1) | 50.7 (36.7-65.9) | |  | 1.3 (0.9-1.7) | 2.2 (1.7-2.9) | 44.1 (32.2-56.7) |  | -0.54 (-0.84--0.24) | -0.78 (-1.09--0.46) |
| Central Europe | 4.3 (3.2-5.6) | 3.2 (2.4-4.1) | 61.1 (44.9-77.1) | |  | 3.7 (2.6-4.9) | 1.6 (1.2-2.1) | 30.2 (21.7-39.8) |  | -2.65 (-2.89--2.41) | -2.78 (-3--2.56) |
| Central Latin America | 0.5 (0.3-0.7) | 0.7 (0.5-1) | 13.9 (9.5-18.4) | |  | 0.6 (0.4-0.9) | 0.3 (0.2-0.4) | 5.7 (3.9-7.7) |  | -3.5 (-3.77--3.24) | -3.46 (-3.75--3.18) |
| Central Sub-Saharan Africa | 0.1 (0-0.1) | 0.5 (0.3-0.7) | 10 (6.2-14.4) | |  | 0.2 (0.1-0.2) | 0.4 (0.2-0.6) | 8.3 (5.2-11.8) |  | -0.7 (-0.75--0.66) | -0.74 (-0.78--0.7) |
| East Asia | 17.5 (12.4-23.6) | 2.5 (1.8-3.3) | 51.7 (37.5-67.6) | |  | 37.1 (26.9-49.3) | 2 (1.4-2.7) | 40.9 (30.2-54.1) |  | -0.78 (-0.92--0.64) | -0.86 (-0.97--0.76) |
| Eastern Europe | 9.1 (6.6-11.8) | 3.5 (2.6-4.5) | 69.3 (50.3-89.4) | |  | 7 (5.1-9.1) | 2 (1.5-2.6) | 41.4 (29.9-53.1) |  | -2.8 (-3.35--2.24) | -2.6 (-3.15--2.05) |
| Eastern Sub-Saharan Africa | 0.3 (0.2-0.4) | 0.5 (0.3-0.7) | 10.7 (7.1-15) | |  | 0.6 (0.4-0.8) | 0.5 (0.3-0.6) | 9.9 (6.4-13.8) |  | -0.21 (-0.26--0.16) | -0.34 (-0.4--0.28) |
| High-income Asia Pacific | 2 (1.5-2.6) | 1.1 (0.8-1.5) | 21.6 (16.1-27.3) | |  | 1.3 (0.9-1.7) | 0.2 (0.2-0.3) | 5.8 (4.2-7.5) |  | -6.21 (-6.47--5.94) | -5.08 (-5.31--4.86) |
| High-income North America | 1.5 (1.1-1.9) | 0.4 (0.3-0.5) | 9.9 (7.6-12.5) | |  | 1 (0.7-1.3) | 0.1 (0.1-0.2) | 4.2 (3.1-5.4) |  | -4.33 (-4.69--3.96) | -3.37 (-3.64--3.09) |
| High-middle SDI | 24.3 (17.9-31.1) | 2.5 (1.9-3.3) | 50.6 (37-64.1) | |  | 29.1 (21.5-37.8) | 1.4 (1.1-1.9) | 29.6 (21.8-38.1) |  | -2.35 (-2.58--2.11) | -2.26 (-2.49--2.03) |
| High SDI | 8.1 (6-10.4) | 0.8 (0.6-1) | 16.3 (12.1-20.4) | |  | 5 (3.7-6.5) | 0.2 (0.2-0.3) | 6.4 (4.8-8.1) |  | -4.56 (-4.82--4.31) | -3.68 (-3.92--3.44) |
| Low-middle SDI | 6.5 (4.5-9) | 1.4 (1-2) | 28.2 (19.7-38.2) | |  | 14.4 (10.2-19) | 1.2 (0.9-1.6) | 24.6 (17.9-31.8) |  | -0.68 (-0.77--0.59) | -0.58 (-0.64--0.52) |
| Low SDI | 1.5 (0.9-2.2) | 0.8 (0.5-1.2) | 16.8 (11-24.5) | |  | 3.1 (2.1-4.3) | 0.7 (0.5-1) | 15.4 (10.6-20.9) |  | -0.54 (-0.6--0.48) | -0.4 (-0.45--0.35) |
| Middle SDI | 16.5 (11.6-21.7) | 2 (1.5-2.7) | 41.4 (29.9-53.3) | |  | 34.6 (25.2-45.4) | 1.6 (1.2-2.1) | 32.9 (24.2-42.1) |  | -0.83 (-0.9--0.75) | -0.82 (-0.87--0.76) |
| North Africa and Middle East | 3.3 (2.4-4.3) | 2.4 (1.7-3.1) | 49.4 (35.6-64.2) | |  | 7.3 (5.4-9.4) | 2 (1.5-2.5) | 42.9 (31.6-54.7) |  | -0.51 (-0.6--0.42) | -0.34 (-0.43--0.24) |
| Oceania | 0 (0-0) | 1.2 (0.8-1.7) | 26.6 (17.4-37.5) | |  | 0.1 (0-0.1) | 1.1 (0.7-1.6) | 26.5 (17.8-37.1) |  | -0.13 (-0.17--0.1) | -0.05 (-0.09-0) |
| South Asia | 5 (3.2-7.2) | 1.3 (0.8-1.9) | 23.1 (15.5-33.5) | |  | 10.9 (7.4-14.8) | 0.9 (0.6-1.3) | 17.8 (12.3-23.8) |  | -1.5 (-1.69--1.31) | -1.23 (-1.36--1.09) |
| Southeast Asia | 3.7 (2.5-5) | 1.9 (1.3-2.5) | 37.5 (26.1-49.1) | |  | 9.2 (6.5-12.3) | 1.9 (1.3-2.5) | 37.1 (26.2-49.1) |  | 0.12 (0.02-0.21) | 0.1 (0.03-0.16) |
| Southern Latin America | 0.7 (0.5-0.9) | 1.5 (1.1-2) | 27.5 (19.8-35.5) | |  | 0.5 (0.4-0.7) | 0.6 (0.4-0.8) | 10.6 (7.7-13.7) |  | -3.49 (-3.72--3.27) | -3.52 (-3.73--3.3) |
| Southern Sub-Saharan Africa | 0.3 (0.2-0.4) | 1.3 (0.9-1.8) | 27.1 (19.4-35.2) | |  | 0.5 (0.3-0.6) | 1.1 (0.7-1.4) | 20.1 (14.2-26.9) |  | -0.76 (-1.14--0.38) | -0.97 (-1.32--0.62) |
| Tropical Latin America | 1.8 (1.3-2.3) | 2.3 (1.7-3) | 45.9 (33.2-58.3) | |  | 1.5 (1-1.9) | 0.6 (0.5-0.8) | 12.4 (8.9-16.1) |  | -4.48 (-4.71--4.25) | -4.64 (-4.89--4.39) |
| Western Europe | 5.1 (3.7-6.7) | 0.8 (0.6-1.1) | 16.2 (12.1-20.3) | |  | 2.3 (1.6-3) | 0.2 (0.2-0.3) | 4.3 (3.2-5.5) |  | -5.32 (-5.58--5.07) | -4.89 (-5.14--4.64) |
| Western Sub-Saharan Africa | 0.4 (0.3-0.6) | 0.6 (0.4-0.8) | 12.7 (8.4-17.8) | |  | 0.7 (0.5-1) | 0.5 (0.3-0.7) | 10.5 (7.3-14) |  | -0.75 (-0.84--0.65) | -0.79 (-0.89--0.7) |

ASMR: age-standardized mortality rate; ASDR: age-standardized disability-adjusted life year rate; EAPC: estimated annual percentage change.

**Supplementary Table 3. SAH burden attributable to secondhand smoke in 1990 and 2019, and its temporal trends from 1990 to 2019.**

| **Characteristics** | **1990** | | |  | **2019** | | |  | **EAPC** | |
| --- | --- | --- | --- | --- | --- | --- | --- | --- | --- | --- |
|  | **Incident cases,**  **n × 10^3^ (95% UI)** | **ASMR per 100,000,**  **n (95% UI)** | **ASDR per 100,000,**  **n (95% UI)** |  | **Incident cases,**  **n × 10^3^ (95% UI)** | **ASMR per 100,000,**  **n (95% UI)** | **ASDR per 100,000,**  **n (95% UI)** |  | **ASMR** | **ASDR** |
| **Global** | 19.9 (13.5-26.3) | 0.5 (0.3-0.7) | 14.5 (9.9-19.1) |  | 13.5 (10-17.7) | 0.2 (0.1-0.2) | 5.4 (3.9-7.1) |  | -4.51 (-4.91--4.1) | -3.99 (-4.32--3.66) |
| **Sex** |  |  |  |  |  |  |  |  |  |  |
| Female | 13.4 (9.3-17.9) | 0.6 (0.4-0.8) | 19 (13.2-25.5) |  | 8.2 (6.1-10.9) | 0.2 (0.1-0.3) | 6.6 (4.7-8.8) |  | -4.79 (-5.19--4.38) | -4.26 (-4.6--3.92) |
| Male | 6.5 (3.3-9.3) | 0.3 (0.2-0.5) | 9.8 (5.3-14) |  | 5.2 (3.8-7.1) | 0.1 (0.1-0.2) | 4.2 (3-5.7) |  | -3.97 (-4.37--3.57) | -3.49 (-3.82--3.17) |
| **Socio-demographic index** |  |  |  |  |  |  |  |  |  |  |
| Low SDI | 0.5 (0.2-1) | 0.2 (0.1-0.4) | 6.5 (2.6-11.7) |  | 0.8 (0.4-1.4) | 0.1 (0.1-0.2) | 4.4 (2.2-7.4) |  | -1.75 (-1.86--1.64) | -1.6 (-1.7--1.5) |
| Low-middle SDI | 3.2 (1.7-4.8) | 0.5 (0.3-0.8) | 14.7 (8.2-22.1) |  | 3.4 (2.1-4.7) | 0.2 (0.1-0.3) | 7.3 (4.7-10.5) |  | -3.25 (-3.57--2.94) | -2.84 (-3.09--2.59) |
| Middle SDI | 9.6 (6.2-12.6) | 1 (0.6-1.3) | 24.3 (16-31.7) |  | 5 (3.6-6.6) | 0.2 (0.1-0.3) | 6 (4.4-7.8) |  | -6.43 (-7.09--5.77) | -5.64 (-6.21--5.07) |
| High-middle SDI | 4.6 (3.3-5.9) | 0.4 (0.3-0.5) | 13.1 (9.5-16.9) |  | 2.9 (2.2-3.8) | 0.1 (0.1-0.2) | 5 (3.7-6.5) |  | -4.28 (-4.55--4.01) | -3.91 (-4.14--3.67) |
| High SDI | 2 (1.5-2.5) | 0.2 (0.2-0.3) | 8 (6-10.2) |  | 1.4 (1-1.7) | 0.1 (0.1-0.1) | 3.5 (2.6-4.5) |  | -3.31 (-3.47--3.16) | -3.15 (-3.3--3) |
| **Region** |  |  |  |  |  |  |  |  |  |  |
| Andean Latin America | 0.1 (0-0.1) | 0.2 (0.1-0.3) | 7.6 (5-11) |  | 0.1 (0-0.1) | 0.1 (0.1-0.1) | 3.5 (2.2-5.1) |  | -2.85 (-3.08--2.62) | -2.81 (-3.01--2.61) |
| Australasia | 0 (0-0) | 0.2 (0.1-0.2) | 5.8 (4.2-7.6) |  | 0 (0-0) | 0.1 (0-0.1) | 2.5 (1.8-3.3) |  | -3.11 (-3.24--2.98) | -3.08 (-3.2--2.96) |
| Caribbean | 0.1 (0-0.1) | 0.2 (0.1-0.3) | 7.4 (4.9-10.4) |  | 0.1 (0-0.1) | 0.1 (0.1-0.2) | 4.8 (3.1-6.8) |  | -1.71 (-1.95--1.48) | -1.73 (-1.99--1.47) |
| Central Asia | 0.1 (0.1-0.2) | 0.2 (0.2-0.3) | 7.8 (5.4-10.4) |  | 0.2 (0.1-0.2) | 0.2 (0.2-0.3) | 7.2 (5.3-9.3) |  | 0.17 (0.01-0.32) | -0.26 (-0.41--0.11) |
| Central Europe | 0.5 (0.3-0.6) | 0.3 (0.2-0.4) | 11.9 (8.8-15.1) |  | 0.4 (0.3-0.5) | 0.2 (0.1-0.3) | 6.4 (4.7-8.5) |  | -2.14 (-2.3--1.98) | -2.44 (-2.6--2.28) |
| Central Latin America | 0.2 (0.1-0.2) | 0.2 (0.1-0.2) | 5.7 (4-7.8) |  | 0.3 (0.2-0.4) | 0.1 (0.1-0.2) | 4 (2.6-5.6) |  | -1.21 (-1.28--1.13) | -1.44 (-1.53--1.34) |
| Central Sub-Saharan Africa | 0 (0-0) | 0.1 (0-0.1) | 2 (0.8-4.1) |  | 0 (0-0.1) | 0 (0-0.1) | 1.6 (0.7-3.4) |  | -0.78 (-0.92--0.64) | -0.71 (-0.83--0.58) |
| East Asia | 11.6 (7.4-15.4) | 1.4 (0.9-1.9) | 34.2 (22.2-45.1) |  | 4.1 (2.9-5.6) | 0.2 (0.1-0.3) | 5.8 (4.1-7.7) |  | -8.02 (-8.88--7.15) | -7.35 (-8.14--6.56) |
| Eastern Europe | 0.6 (0.4-0.7) | 0.2 (0.2-0.3) | 7.9 (5.7-10.3) |  | 0.7 (0.5-0.9) | 0.2 (0.2-0.3) | 8.2 (5.9-10.9) |  | -0.42 (-1.11-0.26) | -0.34 (-0.91-0.24) |
| Eastern Sub-Saharan Africa | 0.1 (0-0.2) | 0.1 (0-0.2) | 3.1 (1.3-6.5) |  | 0.1 (0.1-0.3) | 0.1 (0-0.1) | 2.2 (0.9-4.5) |  | -1.48 (-1.59--1.38) | -1.45 (-1.55--1.34) |
| High-income Asia Pacific | 0.7 (0.5-0.9) | 0.3 (0.3-0.4) | 12.6 (9.2-16.3) |  | 0.4 (0.3-0.6) | 0.1 (0.1-0.2) | 5.5 (4-7.2) |  | -3.7 (-3.8--3.59) | -3.05 (-3.13--2.96) |
| High-income North America | 0.5 (0.4-0.6) | 0.2 (0.1-0.2) | 6.6 (5-8.4) |  | 0.4 (0.3-0.6) | 0.1 (0.1-0.1) | 3.2 (2.4-4.2) |  | -2.68 (-2.9--2.46) | -2.87 (-3.11--2.63) |
| North Africa and Middle East | 0.6 (0.3-0.9) | 0.3 (0.2-0.5) | 10 (5.3-14.7) |  | 0.6 (0.4-0.8) | 0.1 (0.1-0.2) | 4.1 (2.9-5.6) |  | -3.37 (-3.46--3.29) | -3.19 (-3.28--3.11) |
| Oceania | 0 (0-0) | 0.6 (0.3-1.1) | 19.5 (10-32.8) |  | 0 (0-0.1) | 0.5 (0.3-0.8) | 16.2 (8.9-26.6) |  | -0.77 (-0.81--0.73) | -0.58 (-0.62--0.53) |
| South Asia | 2.3 (0.9-4) | 0.4 (0.1-0.7) | 11.2 (4.4-19.1) |  | 3.2 (1.8-4.9) | 0.2 (0.1-0.3) | 7 (4.1-10.6) |  | -2.08 (-2.17--2) | -1.78 (-1.86--1.7) |
| Southeast Asia | 1 (0.7-1.5) | 0.4 (0.2-0.5) | 11.9 (7.6-17) |  | 1.5 (1-2.2) | 0.2 (0.2-0.3) | 7.7 (5-11) |  | -1.57 (-1.61--1.53) | -1.37 (-1.41--1.33) |
| Southern Latin America | 0.2 (0.1-0.2) | 0.4 (0.3-0.5) | 14.6 (10.4-18.9) |  | 0.1 (0.1-0.2) | 0.2 (0.1-0.2) | 6.2 (4.5-8.2) |  | -3.23 (-3.45--3.01) | -3.36 (-3.57--3.15) |
| Southern Sub-Saharan Africa | 0 (0-0) | 0.1 (0.1-0.1) | 3.3 (2.3-4.5) |  | 0 (0-0.1) | 0.1 (0-0.1) | 2 (1.4-2.8) |  | -1.54 (-1.69--1.39) | -1.6 (-1.74--1.45) |
| Tropical Latin America | 0.5 (0.4-0.6) | 0.4 (0.3-0.6) | 16.9 (12-22.3) |  | 0.5 (0.4-0.6) | 0.2 (0.1-0.3) | 6.9 (5.1-9.3) |  | -2.95 (-3.05--2.86) | -3.29 (-3.4--3.18) |
| Western Europe | 0.8 (0.6-1) | 0.2 (0.1-0.2) | 6.1 (4.5-7.8) |  | 0.5 (0.4-0.6) | 0.1 (0.1-0.1) | 2.7 (2-3.6) |  | -2.93 (-3.05--2.81) | -3.03 (-3.15--2.91) |
| Western Sub-Saharan Africa | 0.1 (0-0.1) | 0.1 (0-0.1) | 1.8 (0.8-3.6) |  | 0.1 (0-0.2) | 0 (0-0.1) | 1.2 (0.6-2.4) |  | -1.57 (-1.69--1.45) | -1.41 (-1.53--1.29) |

ASMR: age-standardized mortality rate; ASDR: age-standardized disability-adjusted life year rate; EAPC: estimated annual percentage change

**Supplementary Table 4. Stroke burden attributable to secondhand smoke in 1990 and 2019 and its temporal trends from 1990 to 2019 by nation.**

| **Location** | **1990** | | |  | **2019** | | |  | **EAPC** | |
| --- | --- | --- | --- | --- | --- | --- | --- | --- | --- | --- |
|  | **Incident cases,**  **n × 10^3^ (95% UI)** | **ASMR per 100,000,**  **n (95% UI)** | **ASDR per 100,000,**  **n (95% UI)** |  | **Incident cases,**  **n × 10^3^ (95% UI)** | **ASMR per 100,000,**  **n (95% UI)** | **ASDR per 100,000,**  **n (95% UI)** |  | **ASDR** | **ASMR** |
| Afghanistan | 0.5 (0.3-0.7) | 7 (4.4-9.9) | 189.3 (119.8-269.4) |  | 0.8 (0.5-1.1) | 6 (3.9-8.5) | 154.5 (101-218.8) |  | -0.69 (-0.92--0.46) | -0.88 (-1.15--0.62) |
| Albania | 0.1 (0.1-0.2) | 7 (5.2-9.2) | 135.1 (100.7-172.7) |  | 0.2 (0.1-0.3) | 4.6 (3.1-6.5) | 82.9 (56-117.8) |  | -1.61 (-1.8--1.42) | -1.83 (-2.01--1.65) |
| Algeria | 0.7 (0.5-0.9) | 7 (5-9.3) | 152.8 (109.9-202.7) |  | 1.1 (0.8-1.5) | 3.9 (2.8-5.2) | 82.3 (58.4-109.2) |  | -1.98 (-2.03--1.93) | -2.19 (-2.25--2.14) |
| American Samoa | 0 (0-0) | 5.6 (4-7.1) | 146.5 (105.5-187.2) |  | 0 (0-0) | 3.8 (2.7-5.2) | 105.5 (73.6-142.1) |  | -1.6 (-1.77--1.43) | -1.46 (-1.64--1.29) |
| Andorra | 0 (0-0) | 0.8 (0.5-1.1) | 19 (13-26.6) |  | 0 (0-0) | 0.3 (0.2-0.5) | 9.4 (6.5-12.9) |  | -2.83 (-3.03--2.63) | -2.66 (-2.83--2.49) |
| Angola | 0.1 (0.1-0.1) | 2.5 (1.6-3.5) | 70.6 (46.5-100.4) |  | 0.2 (0.1-0.3) | 1.9 (1.3-2.6) | 50.3 (33.8-71.6) |  | -0.98 (-1.05--0.91) | -1.23 (-1.31--1.16) |
| Antigua and Barbuda | 0 (0-0) | 1.9 (1.4-2.4) | 47 (34.2-60.1) |  | 0 (0-0) | 1.1 (0.8-1.5) | 26.1 (18.4-35.3) |  | -2.23 (-2.52--1.95) | -2.36 (-2.63--2.1) |
| Argentina | 1.2 (0.9-1.5) | 3.7 (2.8-4.7) | 95.1 (71.4-120.6) |  | 0.7 (0.5-0.9) | 1.3 (1-1.7) | 33.2 (24.2-42.8) |  | -4.04 (-4.34--3.73) | -4.19 (-4.48--3.89) |
| Armenia | 0.1 (0.1-0.2) | 5.3 (3.9-6.7) | 112.6 (84-141.9) |  | 0.1 (0.1-0.1) | 2.3 (1.7-3.1) | 50.9 (36.4-66.1) |  | -3.74 (-4.15--3.33) | -3.53 (-3.88--3.18) |
| Australia | 0.2 (0.1-0.2) | 1 (0.7-1.3) | 23.8 (17.9-30.2) |  | 0.1 (0.1-0.2) | 0.3 (0.2-0.4) | 7.6 (5.7-9.8) |  | -4.56 (-4.77--4.35) | -4.08 (-4.29--3.87) |
| Austria | 0.2 (0.1-0.2) | 1.4 (1-1.8) | 34.2 (25.5-43.2) |  | 0.1 (0.1-0.1) | 0.4 (0.3-0.6) | 11.5 (8.6-14.6) |  | -4.58 (-4.85--4.32) | -4.24 (-4.49--3.99) |
| Azerbaijan | 0.2 (0.2-0.3) | 5.1 (3.8-6.5) | 124.4 (91.2-157.3) |  | 0.5 (0.3-0.6) | 6.2 (4.4-8.2) | 125.1 (88.3-165.4) |  | 1.26 (0.84-1.68) | 0.39 (0.03-0.75) |
| Bahamas | 0 (0-0) | 1.8 (1.3-2.4) | 49 (35.5-63.2) |  | 0 (0-0) | 1.2 (0.8-1.6) | 32.1 (22.3-44.3) |  | -1.69 (-1.82--1.55) | -1.6 (-1.75--1.45) |
| Bahrain | 0 (0-0) | 3.1 (2.3-4.1) | 68.4 (50.4-88.5) |  | 0 (0-0) | 1.3 (0.9-1.9) | 27.4 (19.4-37.8) |  | -3.02 (-3.25--2.78) | -3.36 (-3.52--3.2) |
| Bangladesh | 2.8 (2-3.8) | 6.4 (4.4-8.7) | 152.6 (105-205.7) |  | 5.4 (3.6-7.5) | 4.4 (3-6.1) | 98 (65.7-135.9) |  | -0.82 (-1.19--0.45) | -1.11 (-1.38--0.85) |
| Barbados | 0 (0-0) | 1.5 (1.1-1.9) | 37 (26.7-48) |  | 0 (0-0) | 0.8 (0.5-1) | 19.7 (13.5-27.1) |  | -2.73 (-2.98--2.48) | -2.59 (-2.81--2.37) |
| Belarus | 0.4 (0.3-0.5) | 3.2 (2.4-4.1) | 81.6 (60-103.9) |  | 0.4 (0.3-0.5) | 2.3 (1.6-3.2) | 57.8 (40.1-79) |  | -1.87 (-2.3--1.44) | -1.93 (-2.39--1.47) |
| Belgium | 0.2 (0.2-0.3) | 1.4 (1-1.7) | 33.3 (25.4-41.6) |  | 0.1 (0.1-0.1) | 0.4 (0.3-0.6) | 11.4 (8.5-14.5) |  | -4.13 (-4.27--4) | -3.89 (-4.02--3.77) |
| Belize | 0 (0-0) | 1.3 (0.9-1.8) | 34 (23.4-45.6) |  | 0 (0-0) | 1 (0.7-1.4) | 27.4 (19.5-36.5) |  | -1.17 (-1.51--0.83) | -1.16 (-1.49--0.83) |
| Benin | 0 (0-0.1) | 2.3 (1.6-3.2) | 61 (40.2-83.6) |  | 0.1 (0.1-0.1) | 1.7 (1.1-2.3) | 43.7 (28.9-62.6) |  | -1.29 (-1.39--1.19) | -1.29 (-1.39--1.18) |
| Bermuda | 0 (0-0) | 1.4 (1-1.8) | 33.4 (23.9-43.7) |  | 0 (0-0) | 0.5 (0.4-0.7) | 13 (9.2-17.9) |  | -3.5 (-3.73--3.27) | -3.45 (-3.71--3.19) |
| Bhutan | 0 (0-0) | 2.1 (1.4-3.1) | 53.3 (33.9-78.8) |  | 0 (0-0) | 1.2 (0.8-1.6) | 27.8 (18.3-39.4) |  | -2.34 (-2.52--2.16) | -2.57 (-2.75--2.39) |
| Bolivia (Plurinational State of) | 0.1 (0-0.1) | 1.7 (1.1-2.3) | 47.2 (30.6-66.8) |  | 0.1 (0-0.1) | 0.7 (0.5-1.1) | 20.3 (12.3-31.3) |  | -2.97 (-3.32--2.63) | -3.14 (-3.46--2.83) |
| Bosnia and Herzegovina | 0.2 (0.1-0.2) | 4.6 (3.4-5.9) | 102.9 (75.4-131.8) |  | 0.3 (0.2-0.3) | 4.3 (3.1-5.9) | 85 (58.9-116.1) |  | -0.27 (-0.52--0.02) | -0.92 (-1.1--0.74) |
| Botswana | 0 (0-0) | 6.2 (4-8.7) | 142.8 (92.7-201.2) |  | 0.1 (0-0.1) | 4.3 (2.8-6.4) | 96.6 (59.1-145.1) |  | -1.5 (-1.83--1.18) | -1.7 (-2.06--1.34) |
| Brazil | 4.4 (3.3-5.5) | 5 (3.7-6.3) | 132 (98.6-166.8) |  | 3.2 (2.4-4.1) | 1.4 (1-1.8) | 35.5 (26.4-45.5) |  | -4.64 (-4.83--4.45) | -4.77 (-4.97--4.57) |
| Brunei Darussalam | 0 (0-0) | 3.7 (2.7-4.8) | 97.2 (69.6-125.8) |  | 0 (0-0) | 1.2 (0.8-1.5) | 30.3 (22.1-39.3) |  | -4.37 (-4.64--4.11) | -4.37 (-4.68--4.06) |
| Bulgaria | 1 (0.7-1.2) | 8.2 (6.2-10.4) | 188.8 (141.8-237.9) |  | 0.7 (0.5-1) | 5 (3.5-6.9) | 111.6 (77.2-152.6) |  | -2.27 (-2.53--2.01) | -2.45 (-2.72--2.18) |
| Burkina Faso | 0.1 (0.1-0.1) | 1.9 (1.2-2.6) | 50.8 (32.7-72.1) |  | 0.2 (0.1-0.2) | 1.7 (1.2-2.4) | 45.5 (29.5-64.1) |  | -0.19 (-0.25--0.12) | -0.44 (-0.56--0.33) |
| Burundi | 0.1 (0-0.1) | 2.5 (1.6-3.6) | 75 (46.2-109.9) |  | 0.1 (0-0.1) | 1.2 (0.8-1.8) | 35.1 (21.2-52.9) |  | -2.79 (-3.08--2.5) | -3.1 (-3.4--2.79) |
| Cabo Verde | 0 (0-0) | 1.3 (0.9-1.7) | 38.6 (26.8-51.3) |  | 0 (0-0) | 1.3 (0.9-1.8) | 31.8 (22-44.7) |  | -0.66 (-0.93--0.38) | -1.09 (-1.27--0.92) |
| Cambodia | 0.3 (0.2-0.4) | 6.6 (4.6-9) | 172.8 (122.6-231.5) |  | 0.6 (0.4-0.8) | 5.1 (3.6-6.9) | 122.5 (85.8-166.8) |  | -1.1 (-1.22--0.99) | -1.43 (-1.55--1.3) |
| Cameroon | 0.1 (0.1-0.1) | 1.9 (1.3-2.6) | 53.3 (35.8-72.3) |  | 0.2 (0.1-0.3) | 1.4 (0.9-2.1) | 39.6 (24.7-59.2) |  | -1.2 (-1.42--0.98) | -1.13 (-1.36--0.89) |
| Canada | 0.3 (0.2-0.3) | 0.8 (0.6-1) | 22.1 (16.5-27.9) |  | 0.2 (0.1-0.2) | 0.3 (0.2-0.4) | 8.2 (6.1-10.6) |  | -4.4 (-4.73--4.08) | -3.94 (-4.27--3.61) |
| Central African Republic | 0 (0-0.1) | 2.8 (1.8-4.1) | 81.4 (51.6-117.8) |  | 0.1 (0-0.1) | 2.3 (1.4-3.4) | 63.9 (39.6-95.5) |  | -0.84 (-0.95--0.73) | -0.98 (-1.1--0.85) |
| Chad | 0.1 (0-0.1) | 2 (1.3-2.8) | 56.6 (37-79.2) |  | 0.1 (0.1-0.2) | 2 (1.3-2.8) | 53.3 (34.2-76.6) |  | 0 (-0.12-0.13) | -0.1 (-0.23-0.04) |
| Chile | 0.3 (0.2-0.4) | 3.6 (2.6-4.7) | 81.5 (59.3-104.3) |  | 0.3 (0.2-0.4) | 1.3 (1-1.8) | 29.1 (20.9-37.8) |  | -3.36 (-3.53--3.2) | -3.47 (-3.64--3.3) |
| China | 66.8 (48.5-86.6) | 9.1 (6.6-11.9) | 202.3 (148.6-260.6) |  | 83.3 (61-109.6) | 4.5 (3.3-5.9) | 97.6 (71.5-127.2) |  | -2.52 (-2.71--2.33) | -2.66 (-2.82--2.51) |
| Colombia | 0.3 (0.2-0.4) | 1.8 (1.2-2.3) | 44.4 (31.4-57.3) |  | 0.3 (0.2-0.5) | 0.6 (0.4-0.9) | 15.6 (10.3-23) |  | -4.19 (-4.41--3.97) | -4.13 (-4.36--3.89) |
| Comoros | 0 (0-0) | 3.8 (2.1-5.6) | 103.2 (53.7-156.4) |  | 0 (0-0) | 2.5 (1.6-3.6) | 63.2 (40.4-92.2) |  | -1.8 (-2--1.6) | -2.06 (-2.31--1.81) |
| Congo | 0 (0-0) | 2.8 (1.7-3.9) | 78.8 (49.7-109.3) |  | 0 (0-0.1) | 1.8 (1.2-2.6) | 47.3 (30.7-69.9) |  | -1.5 (-1.65--1.35) | -1.8 (-1.95--1.64) |
| Cook Islands | 0 (0-0) | 4.5 (3.1-6) | 121.2 (83.8-162.1) |  | 0 (0-0) | 2.5 (1.7-3.4) | 71 (48.9-97) |  | -2.14 (-2.38--1.91) | -1.89 (-2.14--1.64) |
| Costa Rica | 0 (0-0) | 1.4 (1-1.8) | 32.6 (23.7-42.5) |  | 0 (0-0) | 0.6 (0.4-0.8) | 14.4 (9.9-20.2) |  | -3.66 (-4.15--3.16) | -3.3 (-3.76--2.83) |
| C么te d'Ivoire | 0.1 (0.1-0.2) | 3 (2.1-4.1) | 80.3 (54.8-110.1) |  | 0.3 (0.2-0.4) | 2.6 (1.7-3.5) | 65.8 (42.7-92.8) |  | -0.76 (-1.08--0.44) | -0.92 (-1.24--0.6) |
| Croatia | 0.4 (0.3-0.5) | 6.9 (5.2-8.7) | 152.9 (115-191) |  | 0.2 (0.2-0.3) | 2.6 (1.8-3.6) | 53.4 (37.6-74.2) |  | -3.37 (-3.54--3.2) | -3.71 (-3.87--3.56) |
| Cuba | 0.2 (0.2-0.3) | 2.3 (1.7-2.9) | 60.8 (45.7-78.1) |  | 0.2 (0.2-0.3) | 1.2 (0.8-1.6) | 28.8 (19.8-39.6) |  | -2.71 (-2.92--2.5) | -2.99 (-3.21--2.77) |
| Cyprus | 0 (0-0) | 2.4 (1.8-3.2) | 46.7 (34.2-59.5) |  | 0 (0-0) | 0.8 (0.6-1.1) | 16.2 (12.2-20.7) |  | -4.28 (-4.45--4.11) | -4.22 (-4.43--4) |
| Czechia | 0.6 (0.4-0.8) | 4.4 (3.3-5.7) | 96.7 (71.9-123.5) |  | 0.2 (0.2-0.3) | 1.1 (0.7-1.4) | 24 (17.3-32.3) |  | -5.29 (-5.48--5.09) | -5.06 (-5.23--4.89) |
| Democratic People's Republic of Korea | 1.4 (0.9-1.8) | 9.4 (6.5-12.5) | 226.6 (157.4-300.7) |  | 2.3 (1.7-3.1) | 7.5 (5.4-10) | 184.4 (129.5-250.1) |  | -0.89 (-1.08--0.7) | -0.8 (-0.97--0.63) |
| Democratic Republic of the Congo | 0.2 (0.1-0.3) | 1.4 (0.9-2.1) | 38.7 (23.8-56.9) |  | 0.3 (0.2-0.5) | 1 (0.6-1.5) | 25.5 (14.5-38.7) |  | -1.56 (-1.68--1.44) | -1.55 (-1.66--1.45) |
| Denmark | 0.1 (0.1-0.2) | 1.7 (1.3-2.1) | 42.3 (31.9-53.4) |  | 0.1 (0-0.1) | 0.5 (0.4-0.6) | 11.7 (8.7-15.3) |  | -5.12 (-5.41--4.84) | -5.16 (-5.44--4.88) |
| Djibouti | 0 (0-0) | 4.1 (2.7-5.7) | 102.3 (66.5-142) |  | 0 (0-0) | 3.3 (2.2-4.8) | 77.1 (48.8-113) |  | -0.73 (-0.85--0.62) | -1.01 (-1.11--0.92) |
| Dominica | 0 (0-0) | 1.4 (1-1.9) | 35.4 (25.2-46.8) |  | 0 (0-0) | 1.1 (0.7-1.5) | 25 (16.9-34.8) |  | -0.95 (-1.16--0.73) | -1.23 (-1.44--1.02) |
| Dominican Republic | 0.1 (0.1-0.1) | 1.8 (1.4-2.3) | 49.7 (35.6-64.3) |  | 0.2 (0.1-0.2) | 1.7 (1.1-2.4) | 45.3 (28.9-67.2) |  | 0.07 (-0.15-0.3) | -0.15 (-0.42-0.11) |
| Ecuador | 0.1 (0-0.1) | 1.2 (0.9-1.6) | 35.5 (25-47) |  | 0.1 (0.1-0.1) | 0.5 (0.3-0.7) | 14.4 (9.4-20.6) |  | -3.13 (-3.46--2.8) | -3.35 (-3.65--3.06) |
| Egypt | 1.2 (0.9-1.5) | 4.1 (3-5.4) | 113 (81-143.6) |  | 1.9 (1.2-2.8) | 3 (1.9-4.4) | 83.2 (55.2-120.8) |  | -0.82 (-0.96--0.69) | -0.73 (-0.85--0.61) |
| El Salvador | 0 (0-0) | 0.9 (0.7-1.2) | 26.9 (19-35.7) |  | 0 (0-0) | 0.4 (0.3-0.7) | 12.3 (8.1-18.4) |  | -2.77 (-3.24--2.29) | -2.98 (-3.5--2.45) |
| Equatorial Guinea | 0 (0-0) | 3 (1.8-4.4) | 83.2 (52.2-122.4) |  | 0 (0-0) | 1.4 (0.9-2.1) | 33.5 (20.3-53.1) |  | -3.06 (-3.37--2.76) | -3.56 (-3.91--3.22) |
| Eritrea | 0 (0-0) | 2.2 (1.4-3.3) | 66.4 (42.1-97.5) |  | 0.1 (0-0.1) | 1.8 (1.2-2.7) | 50.2 (32.2-74.1) |  | -0.7 (-0.75--0.65) | -1.02 (-1.07--0.97) |
| Estonia | 0.1 (0.1-0.1) | 4.1 (3-5.2) | 95.7 (68.6-122) |  | 0 (0-0) | 0.8 (0.5-1.1) | 19.7 (13.5-27.2) |  | -7.4 (-8.08--6.71) | -7.01 (-7.66--6.36) |
| Eswatini | 0 (0-0) | 2.5 (1.6-3.5) | 61.2 (38.7-86.7) |  | 0 (0-0) | 1.8 (1.1-2.6) | 42.4 (25.9-64) |  | -0.93 (-1.26--0.61) | -0.91 (-1.3--0.51) |
| Ethiopia | 0.3 (0.2-0.5) | 1.5 (1-2.1) | 44.3 (28.1-64.7) |  | 0.3 (0.2-0.4) | 0.6 (0.4-0.9) | 16.2 (9.8-23.9) |  | -3.43 (-3.6--3.25) | -3.87 (-4.06--3.68) |
| Fiji | 0 (0-0) | 5 (3.4-6.7) | 131 (90-176.6) |  | 0 (0-0) | 3.5 (2.3-4.9) | 90.7 (61.9-127.1) |  | -1.56 (-1.76--1.36) | -1.57 (-1.76--1.38) |
| Finland | 0.1 (0.1-0.1) | 1.2 (0.9-1.5) | 32.8 (24.5-42.3) |  | 0 (0-0.1) | 0.4 (0.3-0.5) | 10.4 (7.6-13.4) |  | -4.2 (-4.39--4) | -4.13 (-4.29--3.96) |
| France | 0.7 (0.6-0.9) | 0.9 (0.7-1.1) | 24.3 (18.2-30.5) |  | 0.4 (0.3-0.5) | 0.3 (0.2-0.3) | 8.2 (6-10.7) |  | -4.54 (-4.72--4.36) | -4.08 (-4.24--3.93) |
| Gabon | 0 (0-0) | 2.5 (1.6-3.5) | 66.4 (43.6-93.7) |  | 0 (0-0) | 1.5 (1-2.2) | 39.1 (24.8-56) |  | -1.78 (-1.95--1.61) | -1.91 (-2.09--1.73) |
| Gambia | 0 (0-0) | 3.4 (2.3-4.8) | 83.2 (54-119.4) |  | 0 (0-0) | 3.3 (2.2-4.5) | 79.7 (52.9-112.5) |  | -0.22 (-0.39--0.05) | -0.23 (-0.42--0.04) |
| Georgia | 0.5 (0.3-0.6) | 7.8 (5.8-9.9) | 176.4 (131.5-224.7) |  | 0.4 (0.3-0.5) | 5.8 (4-7.6) | 128.1 (90.7-168) |  | -0.87 (-1.26--0.48) | -1.08 (-1.49--0.66) |
| Germany | 1.4 (1.1-1.8) | 1.2 (0.9-1.5) | 32 (24.2-40.4) |  | 0.6 (0.5-0.8) | 0.3 (0.3-0.4) | 10.3 (7.6-13.3) |  | -4.48 (-4.82--4.14) | -4.11 (-4.41--3.82) |
| Ghana | 0.1 (0.1-0.1) | 1.4 (0.9-2) | 41.1 (26.2-57.6) |  | 0.2 (0.1-0.3) | 1.3 (0.9-1.8) | 37.1 (24.8-52.5) |  | 0.27 (0.07-0.48) | 0.19 (-0.02-0.39) |
| Greece | 0.5 (0.4-0.7) | 3.6 (2.7-4.5) | 70.4 (53.9-87.2) |  | 0.3 (0.3-0.5) | 1.3 (1-1.6) | 27.8 (20.9-35) |  | -4.19 (-4.48--3.9) | -3.73 (-3.94--3.52) |
| Greenland | 0 (0-0) | 5.9 (4.3-7.6) | 157.3 (114.9-201.8) |  | 0 (0-0) | 2.1 (1.5-2.8) | 57.3 (39.7-77.4) |  | -4.02 (-4.2--3.84) | -3.87 (-4.02--3.73) |
| Grenada | 0 (0-0) | 2.8 (1.9-3.7) | 69.7 (49.3-91.8) |  | 0 (0-0) | 1.4 (1-1.9) | 35.1 (24.2-47.2) |  | -2.29 (-2.56--2.03) | -2.39 (-2.68--2.09) |
| Guam | 0 (0-0) | 3.8 (2.7-5) | 93.5 (65.6-122.8) |  | 0 (0-0) | 2.1 (1.5-2.9) | 68.8 (47.5-92.8) |  | -2.41 (-2.78--2.04) | -1.36 (-1.61--1.11) |
| Guatemala | 0 (0-0) | 1 (0.7-1.3) | 28.4 (19.4-37.4) |  | 0.1 (0-0.1) | 0.7 (0.4-0.9) | 17.5 (11.5-25.2) |  | -2.19 (-2.48--1.9) | -2.44 (-2.75--2.14) |
| Guinea | 0.1 (0.1-0.1) | 2.4 (1.7-3.3) | 63.9 (44.1-88.7) |  | 0.1 (0.1-0.2) | 2.3 (1.5-3.2) | 60.9 (40.7-85.7) |  | 0.2 (0.1-0.29) | 0.18 (0.08-0.29) |
| Guinea-Bissau | 0 (0-0) | 3.4 (2.2-4.7) | 97.2 (62.3-139.5) |  | 0 (0-0) | 2.9 (1.9-4) | 80.1 (53-113.5) |  | -0.29 (-0.38--0.2) | -0.38 (-0.48--0.29) |
| Guyana | 0 (0-0) | 7.7 (5.4-10.5) | 200.9 (140.3-270.4) |  | 0 (0-0) | 3.3 (2.1-4.7) | 82.4 (54.3-118.9) |  | -3.05 (-3.37--2.73) | -3.13 (-3.43--2.83) |
| Haiti | 0.1 (0.1-0.2) | 3.4 (2.1-4.9) | 94.5 (60.6-135.5) |  | 0.1 (0.1-0.2) | 2.1 (1.2-3.3) | 55.1 (32.5-88.2) |  | -1.62 (-1.93--1.31) | -1.73 (-2.06--1.39) |
| Honduras | 0 (0-0.1) | 2.1 (1.5-2.9) | 57.7 (41.4-78.3) |  | 0.1 (0.1-0.2) | 2.4 (1.7-3.4) | 58 (39.4-83.9) |  | 0.55 (0.36-0.74) | -0.01 (-0.17-0.15) |
| Hungary | 0.9 (0.6-1.1) | 6.1 (4.6-7.8) | 147.2 (109.1-185.4) |  | 0.4 (0.3-0.5) | 2 (1.4-2.7) | 46.8 (33.6-62.9) |  | -4.36 (-4.56--4.17) | -4.45 (-4.65--4.26) |
| Iceland | 0 (0-0) | 1.3 (1-1.7) | 32.9 (25.4-41) |  | 0 (0-0) | 0.3 (0.2-0.4) | 8.2 (6.1-10.5) |  | -5.63 (-5.93--5.33) | -5.28 (-5.64--4.92) |
| India | 13.9 (9.5-18.6) | 3.5 (2.4-4.7) | 81 (56.1-107.4) |  | 23.4 (16.2-31.4) | 2.1 (1.5-2.8) | 52.9 (37-70.4) |  | -2.06 (-2.23--1.89) | -1.75 (-1.92--1.59) |
| Indonesia | 5.9 (4.2-7.7) | 6.2 (4.4-8.1) | 166.2 (118.5-216) |  | 13.1 (9-17.4) | 6.7 (4.7-8.9) | 159.7 (110.9-212.7) |  | 0.48 (0.41-0.55) | 0.08 (0.01-0.15) |
| Iran (Islamic Republic of) | 0.8 (0.6-1) | 3.6 (2.6-4.8) | 83 (59.5-108.5) |  | 1.3 (0.9-1.6) | 1.9 (1.4-2.4) | 43.2 (32.2-53.8) |  | -2.45 (-2.62--2.28) | -2.43 (-2.57--2.29) |
| Iraq | 0.5 (0.4-0.7) | 7.2 (5.2-9.5) | 178.1 (128.6-233.6) |  | 1.2 (0.8-1.6) | 5.8 (4-7.7) | 134.8 (92.6-184.5) |  | -0.84 (-0.91--0.77) | -1.09 (-1.18--1.01) |
| Ireland | 0.1 (0.1-0.1) | 1.8 (1.4-2.3) | 41.5 (31.2-52.3) |  | 0 (0-0) | 0.4 (0.3-0.5) | 9.3 (6.7-11.9) |  | -5.86 (-6.15--5.58) | -5.77 (-6.04--5.5) |
| Israel | 0.1 (0-0.1) | 1.2 (0.9-1.5) | 30.6 (22.2-38.6) |  | 0 (0-0.1) | 0.4 (0.3-0.5) | 9.5 (6.8-12.3) |  | -4.93 (-5.22--4.64) | -4.71 (-4.99--4.43) |
| Italy | 1.6 (1.2-2.1) | 1.8 (1.4-2.3) | 42.7 (31.8-53.4) |  | 0.9 (0.6-1.1) | 0.6 (0.4-0.7) | 13.5 (10.2-17) |  | -4.3 (-4.53--4.08) | -4.13 (-4.36--3.89) |
| Jamaica | 0 (0-0.1) | 2.6 (1.9-3.3) | 64.1 (46.8-82) |  | 0.1 (0-0.1) | 1.8 (1.2-2.4) | 42.8 (29.2-60.1) |  | -1.65 (-2.08--1.21) | -1.62 (-2.1--1.14) |
| Japan | 3 (2.2-3.8) | 1.8 (1.4-2.3) | 45.1 (34.1-56.5) |  | 2 (1.4-2.6) | 0.6 (0.4-0.7) | 18.6 (13.8-24.2) |  | -4.33 (-4.48--4.18) | -3.2 (-3.29--3.1) |
| Jordan | 0.1 (0.1-0.1) | 6.3 (4.5-8.3) | 143.7 (102.1-188) |  | 0.1 (0.1-0.2) | 2.4 (1.7-3.2) | 55.1 (39-72.3) |  | -4.12 (-4.52--3.73) | -4.07 (-4.47--3.66) |
| Kazakhstan | 0.7 (0.5-0.9) | 5.8 (4.3-7.5) | 134.8 (98.6-174.6) |  | 0.7 (0.5-0.9) | 4.5 (3.3-6) | 102 (72.6-134.6) |  | -1.34 (-1.69--0.99) | -1.5 (-1.97--1.02) |
| Kenya | 0.2 (0.1-0.2) | 1.9 (1.2-2.7) | 48.8 (31.1-68.6) |  | 0.3 (0.2-0.4) | 1.4 (0.9-2) | 34.3 (21.9-49.6) |  | -1.35 (-1.45--1.24) | -1.36 (-1.51--1.22) |
| Kiribati | 0 (0-0) | 14.5 (10.2-19.8) | 442.6 (307.9-603.6) |  | 0 (0-0) | 12 (8.1-16.6) | 359.6 (242-499.8) |  | -0.92 (-1.14--0.69) | -1.02 (-1.25--0.78) |
| Kuwait | 0 (0-0) | 1.8 (1.3-2.3) | 42.3 (31.8-54.3) |  | 0 (0-0) | 1.5 (1-2) | 34.8 (25.3-45.4) |  | 0.28 (-0.41-0.99) | 0.02 (-0.61-0.65) |
| Kyrgyzstan | 0.2 (0.2-0.3) | 7.1 (5.3-9) | 172.7 (128.5-220.1) |  | 0.2 (0.1-0.3) | 4.6 (3.3-5.9) | 110.5 (80.1-142.6) |  | -1.94 (-2.46--1.42) | -2.04 (-2.56--1.53) |
| Lao People's Democratic Republic | 0.2 (0.1-0.2) | 8.6 (5.7-12) | 217.8 (147.7-305) |  | 0.3 (0.2-0.4) | 6.4 (4.4-8.8) | 153.8 (104.9-212.3) |  | -1.3 (-1.49--1.11) | -1.44 (-1.63--1.24) |
| Latvia | 0.2 (0.1-0.2) | 4.8 (3.5-6.2) | 104.9 (76.7-134.3) |  | 0.1 (0.1-0.1) | 2.3 (1.6-3.1) | 49.5 (34.9-67) |  | -3.42 (-3.81--3.03) | -3.53 (-3.96--3.1) |
| Lebanon | 0 (0-0) | 1.9 (1.4-2.6) | 45.4 (32.9-59.1) |  | 0.1 (0-0.1) | 1.3 (0.8-1.8) | 31.7 (20.8-43.2) |  | -1.18 (-1.33--1.03) | -1 (-1.16--0.83) |
| Lesotho | 0 (0-0) | 3.8 (2.6-5.3) | 90.7 (59.6-125.5) |  | 0.1 (0-0.1) | 5.1 (3.2-7.2) | 121.6 (75.3-176.1) |  | 1.9 (1.49-2.33) | 2.03 (1.58-2.49) |
| Liberia | 0 (0-0) | 1.8 (1.2-2.4) | 47.7 (31.2-66.5) |  | 0 (0-0) | 1.3 (0.8-1.9) | 33.1 (20.6-48.5) |  | -1.25 (-1.35--1.14) | -1.41 (-1.53--1.28) |
| Libya | 0.1 (0-0.1) | 3.3 (2.3-4.5) | 84.8 (58.2-114.8) |  | 0.1 (0.1-0.2) | 3 (2-4.3) | 79.4 (53.5-110.4) |  | -0.27 (-0.43--0.11) | -0.17 (-0.31--0.03) |
| Lithuania | 0.1 (0.1-0.1) | 2.2 (1.6-2.9) | 58.4 (42.4-74.7) |  | 0.1 (0.1-0.1) | 1.4 (1-1.8) | 33.9 (24-45.6) |  | -1.94 (-2.22--1.66) | -2.1 (-2.39--1.81) |
| Luxembourg | 0 (0-0) | 2.1 (1.6-2.7) | 48 (36.6-59.9) |  | 0 (0-0) | 0.4 (0.3-0.6) | 10.5 (7.8-13.7) |  | -5.83 (-5.99--5.67) | -5.56 (-5.72--5.41) |
| Madagascar | 0.3 (0.2-0.3) | 4.7 (3.1-6.3) | 137.9 (92.3-188.5) |  | 0.4 (0.2-0.5) | 3 (1.8-4.6) | 85 (51.6-130.4) |  | -1.93 (-2.08--1.78) | -2 (-2.13--1.87) |
| Malawi | 0.1 (0.1-0.1) | 2 (1.4-2.8) | 55.8 (37.5-78) |  | 0.1 (0.1-0.1) | 1.4 (0.9-2) | 38.1 (23.8-55) |  | -1.58 (-1.73--1.42) | -1.66 (-1.85--1.48) |
| Malaysia | 0.5 (0.4-0.7) | 6.1 (4.4-7.9) | 155.8 (112.6-199.5) |  | 0.8 (0.5-1.1) | 3 (2-4.2) | 75.1 (51.7-105.1) |  | -2.28 (-2.47--2.09) | -2.31 (-2.55--2.06) |
| Maldives | 0 (0-0) | 5.7 (4.1-7.4) | 151.7 (109.1-197.1) |  | 0 (0-0) | 2.1 (1.4-2.8) | 48.2 (34-63.5) |  | -4.09 (-4.38--3.8) | -4.54 (-4.85--4.22) |
| Mali | 0.1 (0.1-0.1) | 2.1 (1.4-2.9) | 58.4 (39.7-80.4) |  | 0.2 (0.1-0.2) | 1.8 (1.2-2.6) | 46.8 (30.7-66.6) |  | -0.44 (-0.54--0.35) | -0.73 (-0.84--0.61) |
| Malta | 0 (0-0) | 1.7 (1.2-2.1) | 39.1 (29.4-49.7) |  | 0 (0-0) | 0.4 (0.3-0.6) | 11.3 (8.4-14.4) |  | -4.67 (-4.8--4.54) | -4.39 (-4.54--4.23) |
| Marshall Islands | 0 (0-0) | 8.1 (5.4-10.9) | 217.8 (144.6-293.4) |  | 0 (0-0) | 6.7 (4.6-9.4) | 192.6 (127.4-268.9) |  | -0.66 (-0.71--0.62) | -0.49 (-0.56--0.41) |
| Mauritania | 0 (0-0) | 2.9 (2-3.9) | 81.1 (54.7-108.2) |  | 0 (0-0) | 1.4 (0.9-2) | 35.9 (23-52.3) |  | -2.51 (-2.63--2.4) | -2.7 (-2.82--2.57) |
| Mauritius | 0 (0-0.1) | 5.9 (4.3-7.7) | 144.1 (103.8-186) |  | 0 (0-0) | 2.2 (1.5-3) | 56.1 (39.3-75.6) |  | -4.51 (-4.98--4.03) | -4.26 (-4.72--3.81) |
| Mexico | 0.6 (0.4-0.8) | 1.4 (1-1.9) | 34.3 (24-45) |  | 0.6 (0.4-0.8) | 0.5 (0.3-0.7) | 13.8 (9.1-19.1) |  | -4 (-4.3--3.69) | -3.67 (-3.96--3.38) |
| Micronesia (Federated States of) | 0 (0-0) | 8.5 (5.7-11.8) | 238.7 (159.7-337.2) |  | 0 (0-0) | 6.8 (4.4-9.9) | 188.8 (114.3-281) |  | -0.82 (-0.93--0.71) | -0.84 (-0.93--0.75) |
| Monaco | 0 (0-0) | 1.5 (1-2) | 33.9 (24.1-45.8) |  | 0 (0-0) | 0.5 (0.4-0.7) | 13.3 (9.3-18.4) |  | -3.71 (-4.08--3.34) | -3.29 (-3.62--2.95) |
| Mongolia | 0.1 (0-0.1) | 6.8 (4.7-9.4) | 194.7 (133.2-270.6) |  | 0.2 (0.1-0.2) | 6.8 (4.6-9.5) | 181.9 (121.4-258.6) |  | -0.26 (-0.85-0.33) | -0.51 (-1.06-0.03) |
| Montenegro | 0 (0-0) | 5.9 (4.4-7.6) | 122.1 (90.2-156.6) |  | 0.1 (0-0.1) | 7.4 (5.3-9.8) | 136.9 (98.7-179.1) |  | 1.04 (0.62-1.46) | 0.47 (0-0.94) |
| Morocco | 0.6 (0.4-0.8) | 4.8 (3.3-6.5) | 115.4 (80.1-153.7) |  | 0.9 (0.6-1.3) | 3.3 (2.2-4.4) | 76.1 (52.3-103.5) |  | -1.47 (-1.59--1.35) | -1.59 (-1.66--1.52) |
| Mozambique | 0.1 (0.1-0.2) | 2.2 (1.4-3.2) | 58.9 (36.4-84.8) |  | 0.3 (0.2-0.4) | 2.5 (1.5-3.7) | 66.3 (39.9-98.5) |  | 0.92 (0.7-1.14) | 0.98 (0.74-1.23) |
| Myanmar | 2.4 (1.7-3.2) | 10.6 (7.4-14.3) | 274.7 (189.7-376.5) |  | 2.6 (1.8-3.6) | 6 (4.2-8.1) | 140.9 (93.4-194.5) |  | -2.24 (-2.38--2.09) | -2.6 (-2.76--2.44) |
| Namibia | 0 (0-0) | 4.1 (2.7-5.6) | 97.8 (62.9-137.7) |  | 0 (0-0) | 2.4 (1.6-3.4) | 53.6 (34.4-78.3) |  | -2.25 (-2.51--1.99) | -2.52 (-2.82--2.22) |
| Nauru | 0 (0-0) | 10.2 (6.9-13.9) | 287.4 (193.5-402.7) |  | 0 (0-0) | 8.5 (5.8-11.8) | 247.5 (164.9-349.1) |  | -0.68 (-1.06--0.31) | -0.58 (-0.99--0.17) |
| Nepal | 0.3 (0.2-0.4) | 3.2 (2-4.7) | 77.5 (48.2-112.2) |  | 0.4 (0.3-0.6) | 2 (1.3-2.8) | 45.3 (28.9-64) |  | -1.7 (-2--1.4) | -1.96 (-2.28--1.63) |
| Netherlands | 0.3 (0.2-0.3) | 1.3 (0.9-1.6) | 30.9 (23.2-39.1) |  | 0.1 (0.1-0.2) | 0.4 (0.3-0.5) | 10.5 (7.8-13.5) |  | -4.58 (-4.94--4.21) | -4.41 (-4.77--4.06) |
| New Zealand | 0 (0-0) | 1 (0.7-1.3) | 26.6 (19.5-33.7) |  | 0 (0-0) | 0.4 (0.3-0.5) | 10 (7.4-12.9) |  | -3.94 (-4.19--3.69) | -3.72 (-3.99--3.46) |
| Nicaragua | 0 (0-0) | 1.6 (1.1-2.2) | 41.5 (29.2-54.8) |  | 0 (0-0.1) | 1.1 (0.7-1.5) | 23.2 (16.4-32.3) |  | -1.97 (-2.21--1.74) | -2.34 (-2.5--2.18) |
| Niger | 0.1 (0-0.1) | 2 (1.3-2.9) | 56 (35.5-80.7) |  | 0.1 (0.1-0.2) | 1.8 (1.1-2.5) | 47.8 (30.7-69.2) |  | -0.47 (-0.63--0.3) | -0.61 (-0.79--0.43) |
| Nigeria | 0.8 (0.5-1.2) | 1.9 (1.1-2.9) | 46.2 (27.7-71.1) |  | 1 (0.7-1.4) | 1.2 (0.8-1.7) | 30.1 (20.1-42.8) |  | -1.76 (-1.94--1.59) | -1.8 (-1.98--1.62) |
| Niue | 0 (0-0) | 7.1 (5-9.5) | 188.5 (131.1-253.2) |  | 0 (0-0) | 5 (3.3-6.9) | 133.7 (89.8-188.4) |  | -1.47 (-1.55--1.4) | -1.44 (-1.53--1.36) |
| North Macedonia | 0.2 (0.1-0.2) | 10.2 (7.5-13) | 209 (156.6-263.7) |  | 0.3 (0.2-0.4) | 9.2 (6.4-12.4) | 168.3 (118-226.8) |  | -0.51 (-0.76--0.26) | -1.02 (-1.24--0.8) |
| Northern Mariana Islands | 0 (0-0) | 5.9 (4.1-7.9) | 156.5 (105.5-213.3) |  | 0 (0-0) | 3.4 (2.4-4.5) | 90.8 (64.2-123.1) |  | -2.2 (-2.45--1.95) | -2.16 (-2.38--1.94) |
| Norway | 0.1 (0.1-0.1) | 1.5 (1.1-1.9) | 35.2 (26.4-44.2) |  | 0 (0-0) | 0.3 (0.2-0.4) | 8.9 (6.6-11.3) |  | -5.72 (-5.95--5.5) | -5.18 (-5.38--4.99) |
| Oman | 0 (0-0) | 3.8 (2.5-5.4) | 86.9 (56.8-125.3) |  | 0 (0-0) | 2.4 (1.7-3.3) | 48.3 (34.6-64.6) |  | -0.96 (-1.25--0.68) | -1.52 (-1.77--1.26) |
| Pakistan | 2.5 (1.7-3.4) | 4.5 (3-6.3) | 112 (76.4-153.9) |  | 3.9 (2.7-5.4) | 3.7 (2.6-5) | 92.1 (64-127.5) |  | -1.14 (-1.44--0.84) | -1.15 (-1.46--0.84) |
| Palau | 0 (0-0) | 6.5 (4.4-9.1) | 184 (120.9-256.3) |  | 0 (0-0) | 5.1 (3.4-7) | 147.4 (99.8-204.4) |  | -0.89 (-0.93--0.85) | -0.77 (-0.8--0.73) |
| Palestine | 0.1 (0-0.1) | 6.6 (4.6-9.1) | 146 (102.3-203) |  | 0.1 (0.1-0.1) | 4 (2.9-5.4) | 87 (62.6-114.2) |  | -1.76 (-1.83--1.69) | -1.86 (-1.92--1.79) |
| Panama | 0 (0-0) | 1.4 (1-1.8) | 32.7 (24.2-42.1) |  | 0 (0-0) | 0.5 (0.4-0.8) | 13.4 (8.9-19.2) |  | -3.53 (-3.74--3.33) | -3.32 (-3.51--3.12) |
| Papua New Guinea | 0.1 (0.1-0.1) | 5.4 (3.2-8.1) | 146.3 (86.6-215.1) |  | 0.3 (0.2-0.4) | 5.5 (3.3-8.2) | 151.3 (91.5-231.3) |  | 0.21 (0.08-0.34) | 0.29 (0.14-0.44) |
| Paraguay | 0.1 (0-0.1) | 2.9 (2.1-3.7) | 73.8 (53.6-95.6) |  | 0.1 (0.1-0.1) | 1.6 (1-2.4) | 40.1 (26.2-60.5) |  | -2.18 (-2.33--2.02) | -2.3 (-2.45--2.15) |
| Peru | 0.1 (0.1-0.2) | 1.1 (0.8-1.5) | 31.3 (21.8-43.3) |  | 0.1 (0.1-0.2) | 0.3 (0.2-0.5) | 10.2 (6.3-15.2) |  | -4.62 (-5.04--4.21) | -4.34 (-4.74--3.93) |
| Philippines | 0.8 (0.6-1.1) | 3.3 (2.4-4.4) | 73.3 (52.6-96.1) |  | 3.1 (2.2-4.2) | 4.2 (2.9-5.6) | 108.4 (75.3-146.3) |  | 1.48 (0.88-2.08) | 2.13 (1.49-2.78) |
| Poland | 1.6 (1.2-2) | 3.7 (2.8-4.7) | 89.3 (68-112.1) |  | 1 (0.7-1.3) | 1.4 (1-1.8) | 32.8 (23.7-43) |  | -3.98 (-4.22--3.74) | -3.96 (-4.17--3.75) |
| Portugal | 0.6 (0.5-0.8) | 4.8 (3.6-6.1) | 99.5 (74.9-125.5) |  | 0.2 (0.2-0.3) | 0.9 (0.7-1.2) | 20 (14.8-25.6) |  | -6.57 (-6.91--6.24) | -6.37 (-6.69--6.05) |
| Puerto Rico | 0 (0-0) | 0.7 (0.5-0.9) | 18.8 (13.8-24.1) |  | 0 (0-0) | 0.3 (0.2-0.5) | 9.6 (6.5-13.7) |  | -3.33 (-3.59--3.07) | -3.02 (-3.27--2.77) |
| Qatar | 0 (0-0) | 2.8 (2-3.9) | 63.3 (44-85.8) |  | 0 (0-0) | 1.4 (0.9-1.9) | 27.2 (18.9-36.6) |  | -2.49 (-2.72--2.25) | -3.13 (-3.31--2.95) |
| Republic of Korea | 1.6 (1.2-2.1) | 6.1 (4.5-7.9) | 146.4 (109.4-184.6) |  | 0.6 (0.4-0.8) | 0.7 (0.5-0.9) | 18 (13.5-23.5) |  | -8.43 (-8.81--8.05) | -7.99 (-8.35--7.64) |
| Republic of Moldova | 0.2 (0.1-0.2) | 4.3 (3.3-5.4) | 107.4 (79.9-135.8) |  | 0.1 (0.1-0.2) | 2 (1.5-2.6) | 52.1 (37.8-68.1) |  | -2.99 (-3.37--2.61) | -2.89 (-3.34--2.45) |
| Romania | 1.6 (1.1-2) | 6.1 (4.5-7.8) | 133.9 (99.4-169.6) |  | 1.4 (1-1.9) | 3.5 (2.5-4.8) | 74.6 (52.7-100.2) |  | -2.65 (-3.09--2.22) | -2.85 (-3.31--2.38) |
| Russian Federation | 8.7 (6.4-11.3) | 5.1 (3.8-6.6) | 115.5 (84.3-147.4) |  | 7.2 (5.2-9.5) | 3.1 (2.2-4) | 75.5 (54.6-99.1) |  | -2.64 (-3.37--1.91) | -2.32 (-3.07--1.57) |
| Rwanda | 0.1 (0.1-0.2) | 3.4 (2.3-4.9) | 103.7 (68.6-147) |  | 0.1 (0.1-0.1) | 1.5 (0.9-2.2) | 39.3 (24.5-59.3) |  | -3.8 (-4.22--3.38) | -4.36 (-4.82--3.9) |
| Saint Kitts and Nevis | 0 (0-0) | 6 (4.4-7.7) | 148.9 (110.3-190.9) |  | 0 (0-0) | 2.2 (1.5-2.9) | 50.9 (33.3-71) |  | -3.91 (-4.25--3.57) | -4.35 (-4.73--3.98) |
| Saint Lucia | 0 (0-0) | 2.7 (2-3.5) | 68.1 (50-89) |  | 0 (0-0) | 1.1 (0.8-1.5) | 29.1 (19.7-39.7) |  | -3.56 (-3.99--3.12) | -3.38 (-3.77--3) |
| Saint Vincent and the Grenadines | 0 (0-0) | 2 (1.4-2.6) | 49.4 (35.3-64) |  | 0 (0-0) | 1.4 (1-1.9) | 35.7 (24.7-47.2) |  | -1.27 (-1.53--1.01) | -1.37 (-1.59--1.14) |
| Samoa | 0 (0-0) | 8.5 (5.8-11.6) | 222 (150.2-305.9) |  | 0 (0-0) | 6.3 (4.3-8.6) | 173.6 (117.8-242.6) |  | -1.07 (-1.13--1) | -0.87 (-0.91--0.83) |
| San Marino | 0 (0-0) | 1 (0.7-1.3) | 22.3 (16.2-29.2) |  | 0 (0-0) | 0.5 (0.3-0.8) | 12.1 (7.6-18.6) |  | -2.36 (-2.52--2.2) | -2.17 (-2.34--1.99) |
| Sao Tome and Principe | 0 (0-0) | 1 (0.7-1.4) | 31.3 (20.8-43.5) |  | 0 (0-0) | 0.9 (0.6-1.3) | 27.1 (17.8-39.9) |  | -0.81 (-1.05--0.57) | -0.86 (-1.18--0.54) |
| Saudi Arabia | 0.3 (0.2-0.4) | 5.2 (3.5-7.1) | 123.1 (82.2-169.2) |  | 0.6 (0.4-0.8) | 3.5 (2.4-4.6) | 86.4 (59.2-115.7) |  | -1.34 (-1.47--1.21) | -1.05 (-1.19--0.9) |
| Senegal | 0.1 (0.1-0.2) | 4.1 (3-5.5) | 103.8 (72.5-139.6) |  | 0.2 (0.1-0.3) | 2.8 (2-3.8) | 68.6 (46.6-93.7) |  | -1.41 (-1.53--1.3) | -1.45 (-1.59--1.31) |
| Serbia | 0.8 (0.6-1) | 8 (5.7-10.4) | 168.7 (123.2-216.2) |  | 0.8 (0.5-1) | 4.9 (3.4-6.6) | 91.7 (64.6-124.4) |  | -2.18 (-2.6--1.75) | -2.78 (-3.23--2.32) |
| Seychelles | 0 (0-0) | 3.5 (2.6-4.6) | 93.4 (67.4-121.3) |  | 0 (0-0) | 2 (1.5-2.7) | 53.4 (38.6-71) |  | -1.75 (-1.95--1.55) | -1.84 (-2.01--1.67) |
| Sierra Leone | 0.1 (0-0.1) | 3.4 (2.2-5.1) | 85.8 (52.9-128.9) |  | 0.1 (0.1-0.2) | 3 (1.9-4.3) | 73.8 (46.3-109.8) |  | -0.3 (-0.46--0.14) | -0.3 (-0.46--0.13) |
| Singapore | 0 (0-0) | 1.4 (1-1.9) | 39.5 (28.4-51) |  | 0 (0-0) | 0.3 (0.2-0.3) | 8.4 (6-10.9) |  | -6.17 (-6.34--6) | -5.63 (-5.84--5.42) |
| Slovakia | 0.2 (0.2-0.3) | 4.1 (3.1-5.3) | 98.5 (73.3-125.3) |  | 0.2 (0.1-0.2) | 1.7 (1.2-2.3) | 38.3 (26.5-52.6) |  | -2.98 (-3.13--2.83) | -3.13 (-3.28--2.98) |
| Slovenia | 0.1 (0.1-0.1) | 3.4 (2.3-4.8) | 80.3 (54.2-112.7) |  | 0 (0-0.1) | 0.8 (0.6-1.2) | 19.7 (13.8-26.7) |  | -4.96 (-5.23--4.69) | -5.12 (-5.35--4.88) |
| Solomon Islands | 0 (0-0) | 13.2 (8.9-18.4) | 376.1 (253.7-532.9) |  | 0 (0-0.1) | 12.9 (9-17.8) | 356.8 (244.4-501.6) |  | -0.05 (-0.11-0.01) | -0.13 (-0.19--0.06) |
| Somalia | 0.1 (0.1-0.1) | 3.5 (2.2-5) | 97.2 (61.6-139.7) |  | 0.2 (0.1-0.3) | 2.5 (1.5-3.7) | 66.8 (39.3-99.8) |  | -1.05 (-1.16--0.93) | -1.3 (-1.42--1.17) |
| South Africa | 0.7 (0.5-0.9) | 3.3 (2.4-4.4) | 89 (64.4-114.9) |  | 0.8 (0.5-1) | 1.9 (1.3-2.5) | 43.4 (30.1-57.3) |  | -2.06 (-2.53--1.59) | -2.61 (-3.05--2.16) |
| South Sudan | 0.1 (0-0.1) | 2.3 (1.5-3.3) | 59.5 (37.8-85.9) |  | 0.1 (0-0.1) | 1.6 (0.9-2.5) | 39.6 (23.1-60.6) |  | -1.17 (-1.29--1.04) | -1.43 (-1.58--1.27) |
| Spain | 1 (0.7-1.2) | 1.8 (1.4-2.4) | 42.9 (32.6-53.7) |  | 0.5 (0.3-0.6) | 0.5 (0.3-0.6) | 11.6 (8.7-14.9) |  | -5 (-5.2--4.81) | -4.66 (-4.84--4.48) |
| Sri Lanka | 0.3 (0.2-0.3) | 2.8 (2-3.8) | 60.8 (43-80.9) |  | 0.3 (0.2-0.5) | 1.4 (0.9-2.1) | 33 (21.7-47.6) |  | -1.99 (-2.22--1.77) | -2 (-2.17--1.83) |
| Sudan | 0.5 (0.3-0.8) | 6 (4-8.5) | 151.7 (101-214.1) |  | 0.6 (0.4-1) | 3.6 (2.4-5.3) | 89.4 (59.9-131.4) |  | -1.88 (-1.94--1.81) | -1.9 (-1.96--1.84) |
| Suriname | 0 (0-0) | 3.9 (2.9-5) | 100 (74.1-128.7) |  | 0 (0-0) | 3 (2.1-4) | 76.7 (53.7-103.3) |  | -1.38 (-1.81--0.95) | -1.45 (-1.84--1.07) |
| Sweden | 0.1 (0.1-0.2) | 0.8 (0.6-1) | 21.2 (15.9-26.9) |  | 0.1 (0-0.1) | 0.3 (0.2-0.4) | 8.3 (6.2-10.7) |  | -3.9 (-4.07--3.72) | -3.61 (-3.78--3.45) |
| Switzerland | 0.1 (0.1-0.2) | 1.1 (0.8-1.4) | 26.2 (20-32.6) |  | 0.1 (0-0.1) | 0.3 (0.2-0.3) | 6.8 (5-8.8) |  | -5.43 (-5.72--5.13) | -5.14 (-5.43--4.85) |
| Syrian Arab Republic | 0.3 (0.2-0.4) | 5.1 (3.5-7) | 134.6 (93.4-186.3) |  | 0.3 (0.2-0.5) | 3.1 (2.1-4.3) | 76.3 (52.2-107) |  | -2.57 (-2.94--2.19) | -2.66 (-3.04--2.27) |
| Taiwan (Province of China) | 0.8 (0.6-1) | 5.4 (4.1-7) | 127.3 (95.5-160.6) |  | 0.4 (0.3-0.6) | 1.1 (0.8-1.6) | 31.7 (22.3-42.8) |  | -5.99 (-6.36--5.61) | -5.25 (-5.59--4.92) |
| Tajikistan | 0.1 (0.1-0.1) | 4.2 (3.1-5.3) | 107.9 (80-136.5) |  | 0.2 (0.1-0.2) | 4.2 (2.9-5.6) | 92.6 (63.9-125.3) |  | 0.05 (-0.23-0.33) | -0.56 (-0.77--0.35) |
| Thailand | 1.1 (0.8-1.5) | 3.5 (2.5-4.6) | 84.4 (59.6-110.2) |  | 1.3 (0.9-2) | 1.4 (0.9-2) | 38.6 (25.8-55.2) |  | -3.88 (-4.13--3.64) | -3.29 (-3.53--3.04) |
| Timor-Leste | 0 (0-0) | 5.1 (3.3-7.2) | 126.3 (82.9-179.5) |  | 0 (0-0.1) | 5.4 (3.6-7.6) | 128.6 (82.6-183.4) |  | 0.23 (0.13-0.34) | 0.05 (-0.07-0.18) |
| Togo | 0 (0-0) | 2.5 (1.7-3.3) | 70.9 (48.4-95.9) |  | 0.1 (0-0.1) | 1.8 (1.2-2.5) | 51.9 (34.5-72.8) |  | -1.11 (-1.15--1.06) | -1.07 (-1.12--1.03) |
| Tokelau | 0 (0-0) | 7.6 (5.2-10.6) | 205.7 (138.9-288.3) |  | 0 (0-0) | 5 (3.4-7) | 139.8 (92.9-196.9) |  | -1.49 (-1.57--1.41) | -1.41 (-1.49--1.34) |
| Tonga | 0 (0-0) | 3.6 (2.5-4.8) | 93.1 (63.2-124.8) |  | 0 (0-0) | 2.9 (2-3.9) | 75 (51.6-103.1) |  | -0.76 (-0.88--0.64) | -0.79 (-0.89--0.69) |
| Trinidad and Tobago | 0 (0-0) | 3 (2.2-3.9) | 72.7 (53.1-94) |  | 0 (0-0) | 1.5 (1-2.2) | 36.6 (23.9-53) |  | -2.78 (-3.01--2.56) | -2.85 (-3.08--2.62) |
| Tunisia | 0.2 (0.1-0.3) | 4.6 (3.2-6) | 98.8 (70.4-130.1) |  | 0.3 (0.2-0.5) | 2.8 (1.8-4) | 62.1 (41.4-87.8) |  | -1.88 (-2.01--1.75) | -1.77 (-1.89--1.66) |
| Turkey | 1.1 (0.8-1.4) | 3.3 (2.3-4.5) | 76.1 (54.4-101.1) |  | 1.6 (1.1-2.1) | 1.9 (1.3-2.5) | 40.8 (29-54.9) |  | -1.4 (-1.81--1) | -1.77 (-2.07--1.47) |
| Turkmenistan | 0.1 (0.1-0.2) | 6.6 (4.9-8.3) | 168.9 (125.5-213.2) |  | 0.3 (0.2-0.4) | 7.1 (5-9.7) | 184.1 (129.8-248.5) |  | 0.37 (-0.02-0.75) | 0.43 (0.04-0.81) |
| Tuvalu | 0 (0-0) | 9.8 (6.7-13.6) | 272 (183.1-369.6) |  | 0 (0-0) | 6.5 (4.5-9.2) | 182.2 (121.9-258.6) |  | -1.43 (-1.46--1.41) | -1.37 (-1.39--1.35) |
| Uganda | 0.1 (0.1-0.1) | 1.6 (1-2.2) | 42.7 (27.1-61.8) |  | 0.2 (0.1-0.3) | 1.2 (0.8-1.8) | 32.8 (21.1-46.7) |  | -1.58 (-1.9--1.27) | -1.72 (-2.08--1.36) |
| Ukraine | 3.2 (2.4-4.2) | 4.7 (3.5-6) | 104.1 (76.7-134.2) |  | 2.3 (1.7-3) | 3 (2.2-4) | 78.9 (56.4-104.2) |  | -2.25 (-2.56--1.95) | -1.7 (-2.03--1.36) |
| United Arab Emirates | 0 (0-0) | 6.7 (4.5-9.8) | 148.4 (99-216.3) |  | 0.1 (0.1-0.2) | 3.6 (2.4-5) | 84.4 (57.1-118.7) |  | -2.4 (-2.9--1.89) | -2.15 (-2.5--1.8) |
| United Kingdom | 1.4 (1-1.8) | 1.6 (1.2-2) | 37.3 (28.7-47) |  | 0.6 (0.4-0.8) | 0.5 (0.4-0.6) | 12.2 (9.1-15.5) |  | -4.68 (-4.93--4.42) | -4.36 (-4.6--4.11) |
| United Republic of Tanzania | 0.3 (0.2-0.4) | 2.4 (1.6-3.4) | 65.1 (42.8-90.2) |  | 0.5 (0.3-0.7) | 2.1 (1.3-2.9) | 51.8 (33.2-74.5) |  | -0.57 (-0.73--0.41) | -0.91 (-1.1--0.72) |
| United States of America | 2.7 (2.1-3.3) | 0.9 (0.7-1.1) | 26.8 (20.7-33.3) |  | 2.2 (1.7-2.8) | 0.4 (0.3-0.5) | 13.2 (9.9-16.8) |  | -3.22 (-3.5--2.94) | -2.87 (-3.11--2.64) |
| United States Virgin Islands | 0 (0-0) | 1.3 (0.9-1.7) | 33.9 (23.8-46.3) |  | 0 (0-0) | 1 (0.7-1.4) | 25.6 (17.9-35.3) |  | -0.73 (-0.94--0.53) | -0.92 (-1.11--0.73) |
| Uruguay | 0.1 (0.1-0.2) | 3.7 (2.8-4.7) | 87.3 (64.7-110.2) |  | 0.1 (0.1-0.1) | 1.6 (1.2-2) | 36.6 (27-46.3) |  | -3.37 (-3.72--3.02) | -3.5 (-3.84--3.16) |
| Uzbekistan | 0.4 (0.3-0.5) | 3.8 (2.7-4.9) | 95.7 (67.9-124.7) |  | 0.8 (0.5-1.1) | 4.6 (3.3-6.3) | 103.6 (71.2-141.1) |  | 0.2 (-0.62-1.03) | -0.31 (-0.96-0.35) |
| Vanuatu | 0 (0-0) | 6.1 (3.9-9) | 179.4 (111-266.5) |  | 0 (0-0) | 5.7 (3.6-8.4) | 173.2 (108.6-256) |  | -0.64 (-0.84--0.44) | -0.55 (-0.75--0.35) |
| Venezuela (Bolivarian Republic of) | 0.2 (0.2-0.3) | 2.3 (1.6-3) | 57.1 (40.8-74.2) |  | 0.4 (0.3-0.6) | 1.4 (0.9-2.1) | 34.5 (21.9-50.4) |  | -2.06 (-2.27--1.86) | -2.18 (-2.4--1.96) |
| Viet Nam | 2.7 (1.8-3.7) | 7.2 (4.7-10) | 163.2 (108.9-223.1) |  | 4.8 (3.3-6.7) | 5.7 (4-7.9) | 125.9 (85.7-172.3) |  | -0.55 (-0.75--0.36) | -0.64 (-0.8--0.47) |
| Yemen | 0.3 (0.2-0.4) | 6.7 (4.2-9.6) | 160.9 (102.8-231.8) |  | 0.6 (0.4-0.9) | 5.2 (3.4-7.3) | 123.9 (82.1-177.5) |  | -1.08 (-1.15--1) | -1.13 (-1.21--1.04) |
| Zambia | 0.1 (0-0.1) | 2.7 (1.8-3.8) | 71.3 (46.2-101.2) |  | 0.2 (0.1-0.3) | 2.8 (1.7-4.1) | 70.2 (42-103.6) |  | -0.02 (-0.16-0.13) | -0.33 (-0.48--0.18) |
| Zimbabwe | 0.1 (0-0.1) | 1.9 (1.3-2.5) | 44.8 (30.8-60.6) |  | 0.1 (0.1-0.2) | 2 (1.4-3) | 51.1 (34.2-74.7) |  | 1.3 (0.87-1.73) | 1.56 (1.09-2.03) |

ASMR: age-standardized mortality rate; ASDR: age-standardized disability-adjusted life year rate; EAPC: estimated annual percentage change.
